# Early Differential Gene Expression in Haemocytes from Resistant and Susceptible *Biomphalaria glabrata* Strains in Response to *Schistosoma mansoni*

**DOI:** 10.1371/journal.pone.0051102

**Published:** 2012-12-26

**Authors:** Anne E. Lockyer, Aidan M. Emery, Richard A. Kane, Anthony J. Walker, Claus D. Mayer, Guillaume Mitta, Christine Coustau, Coen M. Adema, Ben Hanelt, David Rollinson, Leslie R. Noble, Catherine S. Jones

**Affiliations:** 1 Wolfson Wellcome Biomedical Laboratories, Department of Life Sciences, The Natural History Museum, London, United Kingdom; 2 School of Life Sciences, Kingston University, Kingston-upon-Thames, Surrey, United Kingdom; 3 BioSS (Biomathematics & Statistics Scotland) Office, Rowett Institute of Nutrition and Health, Aberdeen University, Aberdeen, United Kingdom; 4 Ecologie et Evolution des interactions, CNRS Université de Perpignan, Perpignan, France; 5 Sophia Agrobiotech Institute, INRA-CNRS-UNS, 06903 Sophia Antopolis, France; 6 CETI (Center for Evolutionary and Theoretical Immunology), Biology, University of New Mexico, Albuquerque, New Mexico, United States of America; 7 School of Biological Sciences, University of Aberdeen, Aberdeen, United Kingdom; Queensland Institute of Medical Research, Australia

## Abstract

The outcome of infection in the host snail *Biomphalaria glabrata* with the digenean parasite *Schistosoma mansoni* is determined by the initial molecular interplay occurring between them. The mechanisms by which schistosomes evade snail immune recognition to ensure survival are not fully understood, but one possibility is that the snail internal defence system is manipulated by the schistosome enabling the parasite to establish infection. This study provides novel insights into the nature of schistosome resistance and susceptibility in *B. glabrata* at the transcriptomic level by simultaneously comparing gene expression in haemocytes from parasite-exposed and control groups of both schistosome-resistant and schistosome-susceptible strains, 2 h post exposure to *S. mansoni* miracidia, using an novel 5****K cDNA microarray. Differences in gene expression, including those for immune/stress response, signal transduction and matrix/adhesion genes were identified between the two snail strains and tests for asymmetric distributions of gene function also identified immune-related gene expression in resistant snails, but not in susceptible. Gene set enrichment analysis revealed that genes involved in mitochondrial electron transport, ubiquinone biosynthesis and electron carrier activity were consistently up-regulated in resistant snails but down-regulated in susceptible. This supports the hypothesis that schistosome-resistant snails recognize schistosomes and mount an appropriate defence response, while in schistosome-susceptible snails the parasite suppresses this defence response, early in infection.

## Introduction

The tropical freshwater snail *Biomphalaria glabrata* is an intermediate host for several digenean trematode parasitic worms, including *Schistosoma mansoni,* the causative agent of human intestinal schistosomiasis. Human schistosomiasis is the most widespread trematode infection affecting around 200 million people, leading to a chronic debilitating disease and up to 200,000 deaths per year, across 75 developing countries [1]. Because of its medical importance, the *B. glabrata/S. mansoni* system has also emerged as a model for studies into multicellular host-parasite co-evolution, driven by reciprocal evolution of host resistance and parasite infectivity and/or virulence [2], [3]. The initial interactions between snail and invading schistosome are considered to define their respective future reproduction and survival; the parasite transforming from a short-lived free-living form in freshwater to a longer-term asexual parasitic stage in the snail hosts. If the snail cannot suppress and eliminate the invading schistosome quickly it risks parasitic castration (reviewed in [4]) followed by early death [5], [6]. The initial molecular interplay between snails and schistosomes is complex and there exists an urgent need to determine the principal pathways controlling this response, since identifying those factors involved in the intricate balance between the snail internal defence system (IDS) and trematode infectivity mechanisms that determine the success or failure of an infection (reviewed in [7]–[9]) may provide insight into approaches to disrupt the parasitic infection in the snail and break transmission. Furthermore, by understanding the basis of compatibility and the mechanisms underlying snail susceptibility to schistosome infection, the levels of compatibility in field situations can be assessed, leading to enhanced understanding of transmission dynamics which could ultimately inform control strategies.

Susceptibility of *B. glabrata* to *S. mansoni* is a heritable trait [10], with both snail and parasite genes influencing the outcome of infection [11]. In incompatible interactions, the schistosome fails to recognize, penetrate or develop within the snail, or may be destroyed by the IDS; such killing is mediated by haemocytes, ‘macrophage-like’ defence cells, encapsulating and eliminating non-compatible parasites [12]. The “schistosome-resistant” phenotype is defined as individuals or strain refractory to infection by a normally compatible schistosome strain. To establish an infection in a compatible strain, the schistosome larva must prevent the snail from detecting and/or eliminating it. Two hypotheses are that either the parasite remains undetected by the host and therefore no defence response is mounted [13], [14], or that the parasite is able to interfere with or suppress the host response to enable it to establish an infection [15]–[17]. Haemocyte-derived molecules thought to be key regarding snail defence to schistosomes include a diverse family of secreted lectins called fibrinogen-related proteins (FREPs), co-determinants of resistance as shown by RNAi knockdown [12], [18]–[20] that form complexes with schistosome mucins [21]–[23]; and lysosomal enzymes, and reactive oxygen/nitrogen intermediates [8], [23]–[25] which facilitate killing of the parasite. A cytosolic copper/zinc superoxide dismutase (SOD1) has also been associated with the schistosome-resistant phenotype [26], [27]. Moreover, the snail host oxidant response to schistosome infection has been investigated from the perspective of molecular co-evolution, through evaluation of reciprocal anti-oxidant responses of *S. mansoni* [28]; parasite anti-oxidant capacities appear to match closely host haemocyte oxidant responses in sympatric *B. glabrata/S. mansoni* combinations, highlighting the importance of oxidant production by resistant phenotype haemocytes [28]. Migration and recognition/adhesion of haemocytes to transforming miracidia/developing sporocysts are also likely important determinants of the resistance response. Integrin-like cell surface receptors [29] are known to regulate haemocyte adhesion and motility [30]–[32] and a tandem-repeat galectin has been found to bind haemocytes and the tegument of *S. mansoni* sporocysts [33] making it a candidate anti-schistosome pattern recognition receptor. Interestingly, *S. mansoni* excretory-secretory products (ESPs), released by transforming miracidia, have been shown to suppress extracellular signal-regulated kinase (ERK) signalling in haemocytes of susceptible, but not resistant *B. glabrata* [34] demonstrating that in compatible hosts, schistosomes can interfere with pathways that regulate snail haemocyte defence responses such as nitric oxide production [25]. Finally, knock-down of the recently-characterized *B. glabrata* cytokine Macrophage Migration Inhibitory Factor (BgMIF) was shown to reduce encapsulation of *S. mansoni* sporocysts *in vitro* and increase mother sporocyst survival *in vivo* [35]. Hence, the complex nature of the snail haemocyte defence response to schistosomes, outlined above, makes analysis of global gene expression a vital component of research aimed at elucidating the array of underlying mechanisms of snail-schistosome compatibility.

**Table 1:** *Biomphalaria glabrata* gene-specific primers used to amplify specific gene fragments included on the microarray.

Code	Acc No	Gene		Primer sequence
BgB	AB210096	Dermatopontin 1	F	GGTTATGCCAATGACTTCGGAC
			R	GATTGACTTGCTCGCTCACG
BgG	AF179902	Integrin interactor protein	F	CCTTGGGAATGTCATTGCTTG
			R	GACCATTCCACCCTGATTGC
BgI	AF302260	Serine protease B	F	CTAAGATACGGTGCTGGCTCG
			R	GCGTAGACACCTGGTCTGCC
BgK	AY026258	Thioredoxin peroxidase	F	CACTCACCTTGCATGGACTAATG
			R	CAAGCGCAGTGTCTCATCAAC
BgP	AY678119	Type 2 cystatin	F	CAAAATTGTCCACGCCACATC
			R	GATGGTGTTCCCTGTAGTTGGG
BgQ	DQ087398	guanine nucleotide-binding protein Rho	F	GGCAGCAATACGTAAGAAGCTTG
			R	GCTGTGTCCCATAAGGCTAGTTC
BgSOD	AY505496	Cu/Zn superoxide dismutase	F	GGTGATGATGGTGTTGCTGA
			R	GATACCAATGACACCACAAGCTAA

F-forward facing primer. R- Reverse facing primer.

Both a cDNA microarray [36] and a specific stress/immune gene selected oligo array [20], [37], [38] have been employed previously for gene expression analysis in *B. glabrata*. This widely used method of global gene expression analysis involves hybridizing reverse-transcribed cDNA to the array to indicate relative gene expression for each arrayed gene or EST; this approach does not rely on prior knowledge of candidate genes or mechanisms in advance [39]. We previously developed a 2****K cDNA microarray for *B. glabrata* [36], using randomly sequenced ORESTES-derived expressed sequence tags (ESTs) of various snail tissues including haemocytes [40], together with sequences derived from earlier differential gene expression analyses associated with schistosome resistance in snails using differential display (DD) [41]–[43] and suppression subtractive hybridization (SSH) [44]. We have therefore included many genes on the array which may be involved in snail parasite interactions, without making *a priori* assumptions of their functions based on homology to genes characterised in other organisms. Previous experiments using this microarray to compare gene expression *in vivo* in haemocytes from schistosome-exposed *B. glabrata* strains exhibiting resistant or susceptible phenotypes identified more than 90 strain-specific differentially expressed genes [36]. Unlike techniques such as qPCR, the strength of microarray analysis is that it provides a global view of changes in gene expression through simultaneous comparison of large numbers of genes, indicating cellular pathways and processes involved in response to parasite challenge. The 2****K microarray was significantly expanded for use in this study, with the addition of more than 3****K ESTs.

With the aim of identifying snail strain differences in early haemocyte responses to schistosomes, we furthered past approaches towards defining genes and pathways involved in host defence responses by the first use of gene set enrichment analysis (GSEA) [45] and FatiScan [46] in snail-schistosome interaction studies, using the most comprehensive *B. glabrata* cDNA microarray to date. A key question was to determine if strain specific responses to parasite exposure were at an early stage post exposure and to assess if the parasite was influencing (suppressing) the normal snail defence response in the susceptible strain. During invasion of the snail host, molecules are released from the miracidial penetration glands and within 1 hour the ciliated epidermal plates covering the miracidium are released allowing the parasite to transform into a post-miracidium that lacks a protective surface [47]; other ESPs are also released from the schistosome during such early post-embryonic development and ESPs can modulate kinase signalling [34] and protein expression [48] in *B. glabrata* haemocytes *in vitro* within a similar time frame. Thus, 2 hours post-exposure was selected as an appropriate time frame for our investigation to compare haemocyte gene expression changes in both snail strains in the context of schistosome invasion and early schistosome development in the host. The transcriptomic responses of these schistosome-resistant and -susceptible phenotypes to *S. mansoni* provide novel insights into the nature of early stage interactions that are likely to define trematode resistance and susceptibility.

## Methods

### Microarray Construction

In addition to the 2053 ORESTES, SSH and DD clones printed on the 2****K *B. glabrata* cDNA microarray [36], a further 3174 were available from ORESTES libraries [14], [40], and a haemocyte cDNA library [49]. Selected clones, supplied spotted onto Whatman FTA^TM^ cards, were prepared by eluting the DNA from small discs punched from the card using Multiscreen PCR filter plates (Millipore, Billerica, USA); the resultant DNA was used for 100 µl PCRs containing 1×NH_4_ reaction buffer (Bioline, London, UK), 2.5 mM MgCl_2_, 0.2 mM dNTP, 0.2 µM each M13 forward and reverse primers and 0.025 U/µl PCR *Taq* polymerase (Bioline). Cycling conditions were: 94°C for 2 min, then 35 cycles of 94°C for 30 s, 58°C for 30 s and 72°C for 90 s, then a single cycle of 10 min at 72°C. We specifically included several genes implicated in the defence response of resistant snails, including FREP2 [50]; Cu/Zn superoxide dismutase (SOD) [26], [27] and Mn SOD [51]. The array also contained antioxidant genes such as thioredoxin peroxidase, peroxiredoxin 6, peroxinectin, peroxidasin and dual oxidase I and genes for cell signalling proteins including nuclear factor-kappa B (NF-κB), focal adhesion kinase (FAK) and I-kappa-B kinase complex associated protein (IKAP). PCR products were amplified for 7 specific *B. glabrata* genes (chosen as the sequences were available on GenBank, but not already represented in the ESTs from SSH, ORESTES or cDNA library), using primers designed from their sequences (Table 1). For all clones, 2 µg PCR product was transferred to 384-well plates (Molecular Devices (Genetix), New Milton, Hampshire, UK) using a Microlab Star robotic work-station (Hamilton Robotics, Birmingham, UK). A total of 5234 cDNA clones (50–200 ng/µl) were selected for printing. Controls were also included: yeast tRNA (250 ng/µl); *B. glabrata* genomic DNA from snail strains (NHM laboratory code) NHM3017 (derived from BS90 [52]), NHM1742, NHM3036 (derived from BB02 [53]) (200 ng/µl) and genomic DNA from *Biomphalaria tenogophila, Bi straminea, Bi pfeifferi, Bi alexandrina, Bulinus globosus* and *Bu truncatus*; pGem (purified vector with no insert) (75 ng/µl), two specific genes, (ribosomal 18s and cytochrome oxidase I) amplified from *S. mansoni* and blanks containing spotting buffer only. 15 µl aliquots were transferred to a further 384-well plate and 5 µl 4× spotting buffer (600 mM sodium phosphate; 0.04% SDS) added. The clones were printed (in duplicate) in 16 sub-arrays (4 columns×4 rows), with 26×26 clones in each sub-array on aminopropyl silane coated glass slides (Corning® GAPS™ II), at the Microarray Facility, Department of Pathology, University of Cambridge, UK; this array is the second generation *B. glabrata* microarray. Microarrays were processed by baking for 2 h at 80°C and UV cross-linking at 600 mJ. (GenePix Array List (GAL) file: NHM-ABDN B.glabrata 5****K v1 ArrayExpress Archive accession: Array A-MEXP-1401). Differential expression of haemocyte genes using this *B. glabrata* cDNA microarray technology has previously been confirmed by quantitative qPCR [36] and the array was found to deliver robust results in our hands.

### Snail Material and Parasite Exposure

**Figure 1:**
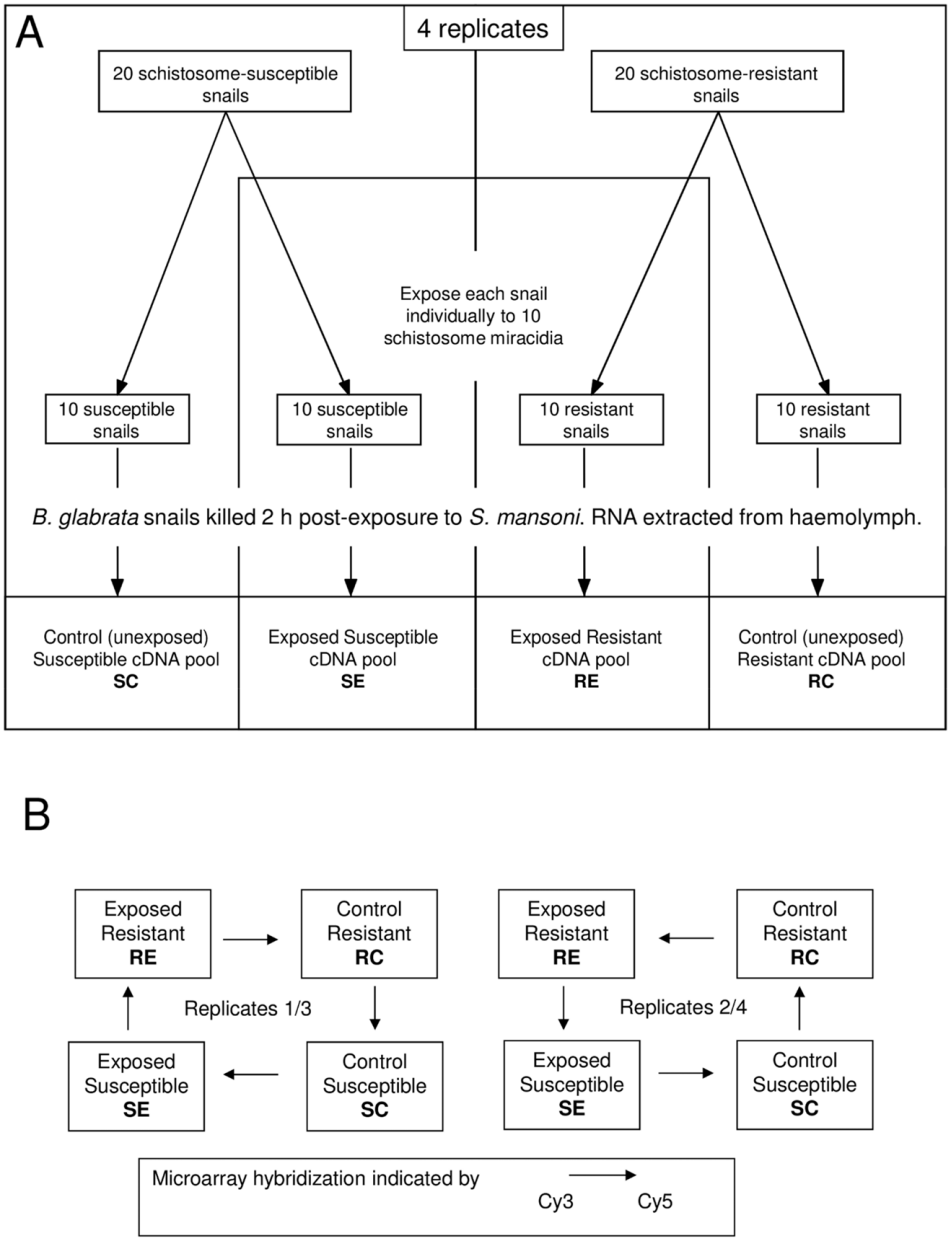
Experimental design for the simultaneous comparison of haemocyte gene expression in *Biomphalaria glabrata* strains upon exposure to *Schistosoma mansoni*. A. Resistant (R) and susceptible (S) strains of *B. glabrata* were exposed to *S. mansoni* (E) or kept unexposed as controls (C) B. Microarray hybridizations: 16 array hybridizations in double loop design with dye swaps were performed.

Four replicate experiments were performed (Fig. 1A). For each replicate, 20 adult *B. glabrata* from the susceptible strain (NHM1742) and 20 from the resistant strain (NHM3017) were maintained overnight in autoclaved snail water containing 100 µg/ml ampicillin. 10 snails from each strain were exposed individually to 10 *S. mansoni* miracidia (Belo Horizonte strain), while 10 were kept in identical conditions, but not exposed to miracidia. Snails were killed swiftly by decapitation 2 h post-exposure, and the exuded haemolymph collected. Haemolymph was pooled for each sample group (resistant exposed (RE), resistant control (RC), susceptible exposed (SE), susceptible control (SC)) and haemocytes pelleted by centrifuging at 10,000×*g* for 20 min at 4°C. The lymph was then removed and the haemocyte pellet frozen in liquid nitrogen and stored at –80°C.

### Microarray Hybridization

Total RNA was extracted from pooled haemocytes, using the SV RNA extraction kit (Promega UK Ltd, Southampton, UK) according to the manufacturer’s protocol. This kit includes DNAse treatment to eliminate genomic DNA contamination. cDNA was synthesized from 100 ng total RNA using the SMART PCR [54] cDNA synthesis kit (BD Biosciences, Oxford. UK) according to the manufacturer’s instructions and labelled with both Cy3 and Cy5 in 2 separate reactions using the BioPrime DNA labeling system (Invitrogen, Paisley, UK). 16 microarray hybridizations were carried out as described previously [36] using a loop design with dye swaps (Fig. 1B). The loop design [55] allowed direct comparison of results from resistant and susceptible snails and control and exposed snails, by comparing, directly on the arrays: i) control and parasite-exposed snails of both the resistant snail line and of the susceptible snail line (4 replicates, 2 with Cy dyes in one orientation and 2 swapped over) and ii) resistant and susceptible snail lines both for control snails and parasite-exposed snails (again with 4 replicates, 2 labelled in one orientation, 2 in the other).

### Microarray Scanning and Analysis

Microarray slides were scanned sequentially for each Cy dye, at 10 µm resolution using an Axon GenePix 4100A scanner (Molecular Devices (UK) Ltd, Wokingham, UK). Photo multiplier tube values were adjusted to give an average intensity ratio between channels of approximately 1. Spot finding and intensity analysis was carried out using GenePix Pro 5.0. Data from these microarray experiments have been deposited with ArrayExpress: Experiment E-MEXP-1882. 16 GenePix output files were analysed using LIMMA (Linear models for microarray data, Bioconductor [56]). Print-tip loess normalization was used for within-array normalization [57] including log-transformation of the gene intensities. Moderated t-statistics were employed to assess change significance and a moderated f-statistic was used to test whether all contrasts were zero simultaneously, that is, whether there was no difference between strains before or after exposure or whether a gene showed an overall effect [58]. Within array duplicates were averaged and showed a good correlation of 0.84. One array displayed weak hybridization and was thus removed from the analysis. LIMMA was also used to define and test for certain contrasts, e.g. the difference of the fold change susceptible/resistant strains between the exposed and control groups (p-values were adjusted for multiple testing using the false Benjamini-Hochberg method [59], which controls the false discovery rate (FDR)).

### Functional Analysis of Genes

Cluster analysis was performed in SeqTools (http://www.seqtools.dk/) using BlastN score values. Basic Local Alignment Search Tool (BLAST) searches, GO (gene ontology) and KEGG (Kyoto Encyclopaedia of Genes and Genomes) annotation and Interpro scans were performed using Blast2GO [60]. An annotation file was generated for the *B. glabrata* microarray (File S1).

### Gene Set Enrichment Analysis

Gene set analysis for the data was performed using the Bioconductor function geneSetTest, which is available within the LIMMA library. The analysis was restricted to those GO terms that had at least 5 genes corresponding to them on the array (333 sets/terms overall). For each of the 5 comparisons (REvSE, RCvSC, REvRC, SEvSC and RE/SEvRC/SC) we tested whether the calculated p-values were more significant for the Gene Set/GO-term in question than for a random selection of genes to generate a Gene Set p-value for each combination of GO-Term and each comparison, and an average p-value across all the genes for each comparison. P-values were adjusted for multiple testing using the Bonferroni method of correction.

### FatiScan

FatiScan ([46], [61], Babelomics: http://babelomics.bioinfo.cipf.es) was employed to identify significant asymmetrical distributions of biological labels (such as GO terms) associated with the ranked genes (based on fold change) for each comparison (REvRC, SEvSC, RCvSC and REvSE), using custom *B. glabrata* microarray annotations generated by Blast2Go.

## Results

### Annotation of Array Genes

**Figure 2:**
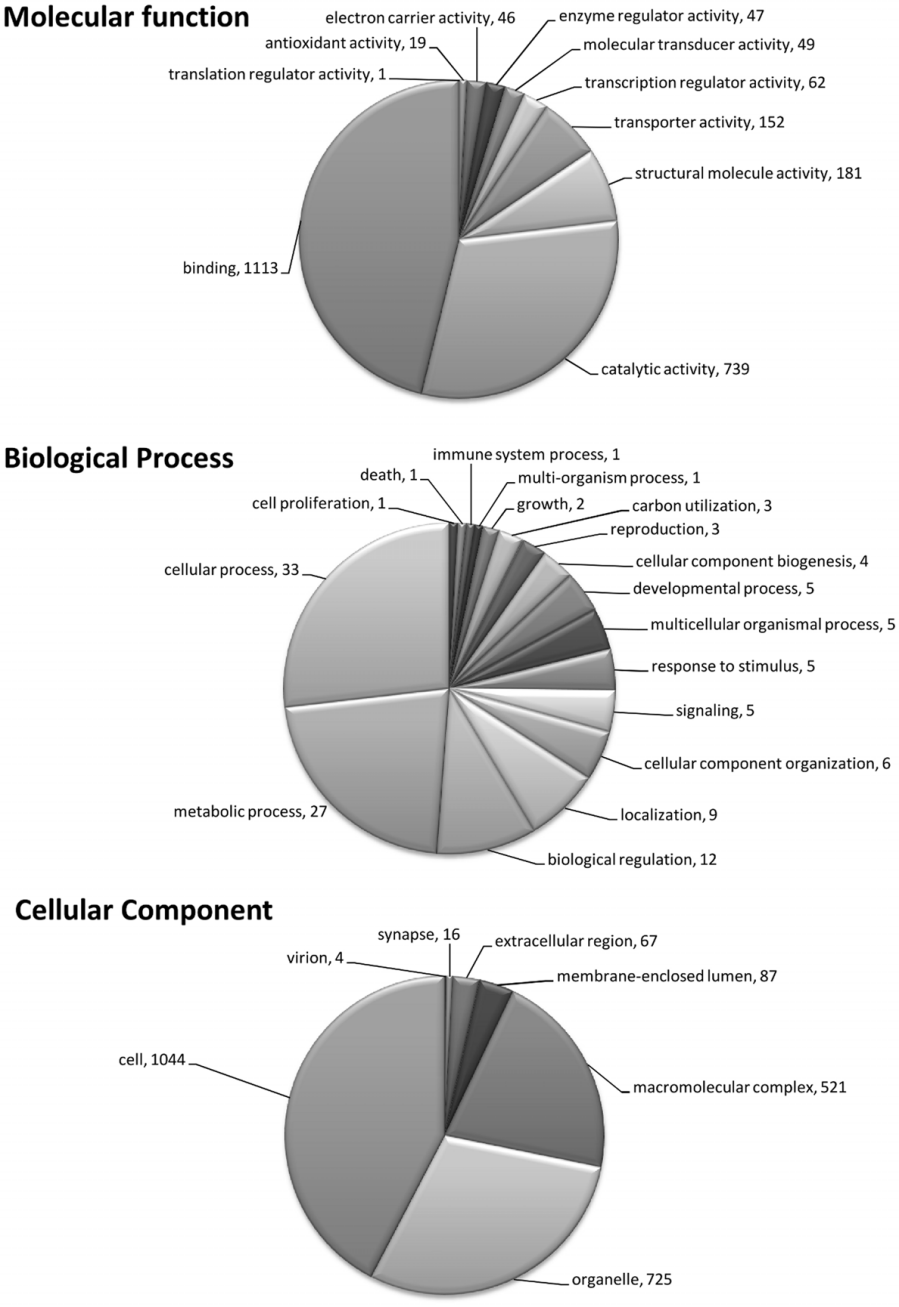
Graphical representation of annotation for version 2 of the *B. glabrata* microarray. The pie charts illustrate the number of genes in each GO assignment for molecular function, biological process and cellular component, for those genes with known functions. Genes that were not assigned are not represented here and each individual sequence may have more than one assignment.

Genes with significant homology to previously characterized genes have GO terms assigned to them on the basis of that homology (Fig. 2). This produced a reference set of annotations associated with the sequences of the genes spotted on the 5****K *B. glabrata* microarray (File S1).

### Haemocyte Genes Differentially Expressed in Response to Parasite Exposure

**Figure 3:**
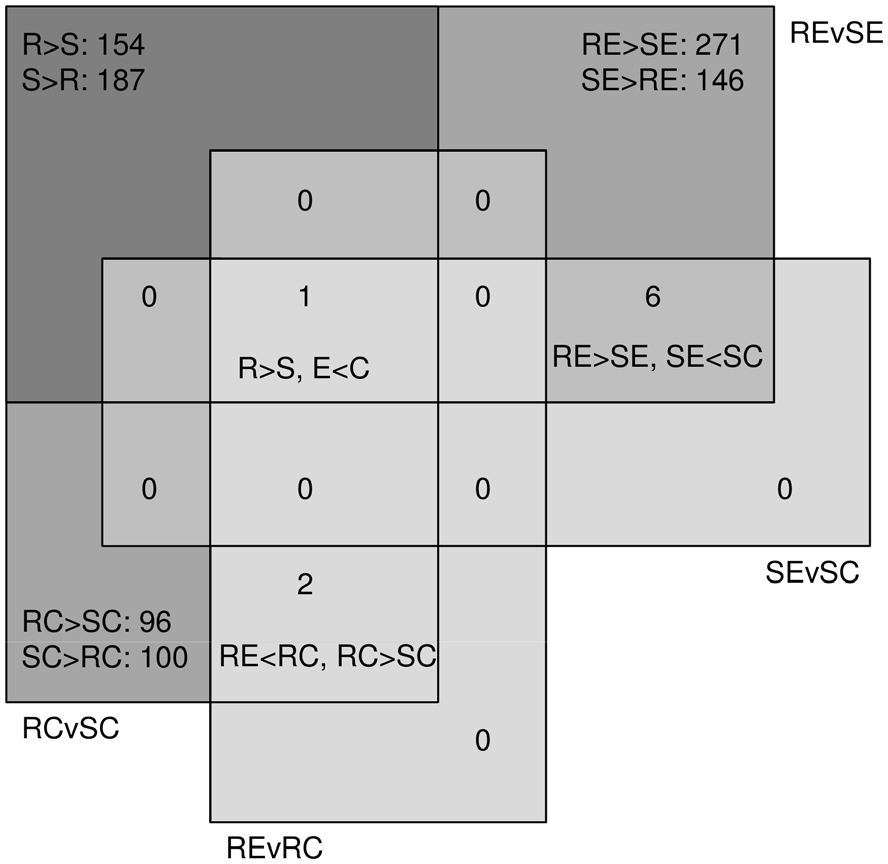
Significant differentially expressed *B. glabrata* haemocyte genes identified from microarray comparisons. The Venn diagram shows the number of identified significantly differentially expressed genes in each category. Some genes were identified which were differentially expressed in more than one comparison and hence lie in the overlapping regions of the diagram. Key to symbols: R, resistant *B. glabrata*; S, susceptible *B. glabrata*; E, *S. mansoni* exposed snails; C, control snails. > greater than, < less than.

mRNA from haemocytes of both control and schistosome-exposed resistant and susceptible snails was compared using the 5****K *B. glabrata* microarray (these data are available at ArrayExpress Archive accession: Array A-MEXP-1401). From the analysis we identified genes that were differentially expressed between control and parasite exposed snails in both strains and between resistant and susceptible strains both for control and parasite exposed snails. The numbers of identified, and the classes in which they demonstrated differential expression, are summarized in the Venn diagram (Fig. 3). Firstly, genes expressed in haemocytes sampled 2 h post exposure to *S. mansoni* miracidia, were compared to unexposed controls to investigate the initial response of each snail strain to the parasite. Analysis of differential gene expression from the microarray identified 9 genes demonstrating a significant difference (p<0.01) in intensity between the compared samples in each category, with some genes located in more than one category (Fig. 3; see also Table 2). One gene (CV548474: unknown) was identified as having significantly higher expression in resistant snails before and after parasite exposure (R>S), and this same gene was also found to be down-regulated in both strains after exposure (E<C). Two genes (EW997021 and EW997112, both unknown, Table 2) were identified which showed significantly higher expression in the resistant control snails (RC>SC) and were down-regulated in the resistant snails after exposure (RE<RC). Additionally, 6 genes were shown to be significantly down-regulated in susceptible snails after infection (SE<SC), which were expressed less in susceptible exposed compared with resistant exposed snails (RE>SE); these included 3 unknown genes, 2 myosin II heavy chain genes and 1 alpha actin gene (Table 2).

**Table 2:** Genes identified as differentially expressed upon exposure to *S. mansoni* in resistant and susceptible *B. glabrata* strains.

Acc No	Name	Organism	Blast match Acc No	E value	fc(RE/SE)	fc(RC/SC)	fc(RE/RC)	fc(SE/SC)
EW997021	Unknown				2.67	**3.81**	**−5.89**	**−**4.12
EW997112	Unknown				2.21	**2.78**	**−4.32**	**−**3.43
CV548474	Unknown				**3.10**	**3.19**	**−5.58**	**−5.43**
EW997519	Unknown				**1.74**	1.27	**−**1.94	**−2.66**
EW997424	Unknown				**3.86**	1.41	1.15	**−2.37**
EW997386	myosin II	*Nasonia vitripennis*	XP_001607303	5.45E−35	**3.02**	1.16	1.18	**−2.21**
EW997539	Unknown				**2.49**	**−**1.04	1.13	**−2.29**
EW997462	myosin II	*Placopecten magellanicus*	2EC6-A	3.65E−75	**2.43**	**−**1.01	1.09	**−2.24**
EW997421	alpha 2 actin	*Dicentrarchus labrax*	ACN66629	1.74E−28	**2.22**	**−**1.02	1.09	**−2.07**

fc – fold change; fc figures in **bold** indicate a significant difference. Resistant control (RC); susceptible control (SC); resistant exposed (RE); susceptible exposed (SE).

### Differences between Schistosome-resistant and Schistosome-susceptible Strains

Secondly, comparison of haemocytes from schistosome-resistant and schistosome-susceptible snails (Fig. 3) revealed large numbers of genes to be differentially expressed between strains, before (196, comprising 96 resistant-specific, and 100 susceptible-specific transcripts) and after exposure (417, comprising 146 in susceptible and 271 in resistant snails). Additionally, 341 genes were differentially expressed regardless of schistosome infection (Fig. 3); without exception, all 187 susceptible-specific genes remained such either before or after exposure, as did all 154 resistant-specific genes (data not shown).

**Figure 4:**
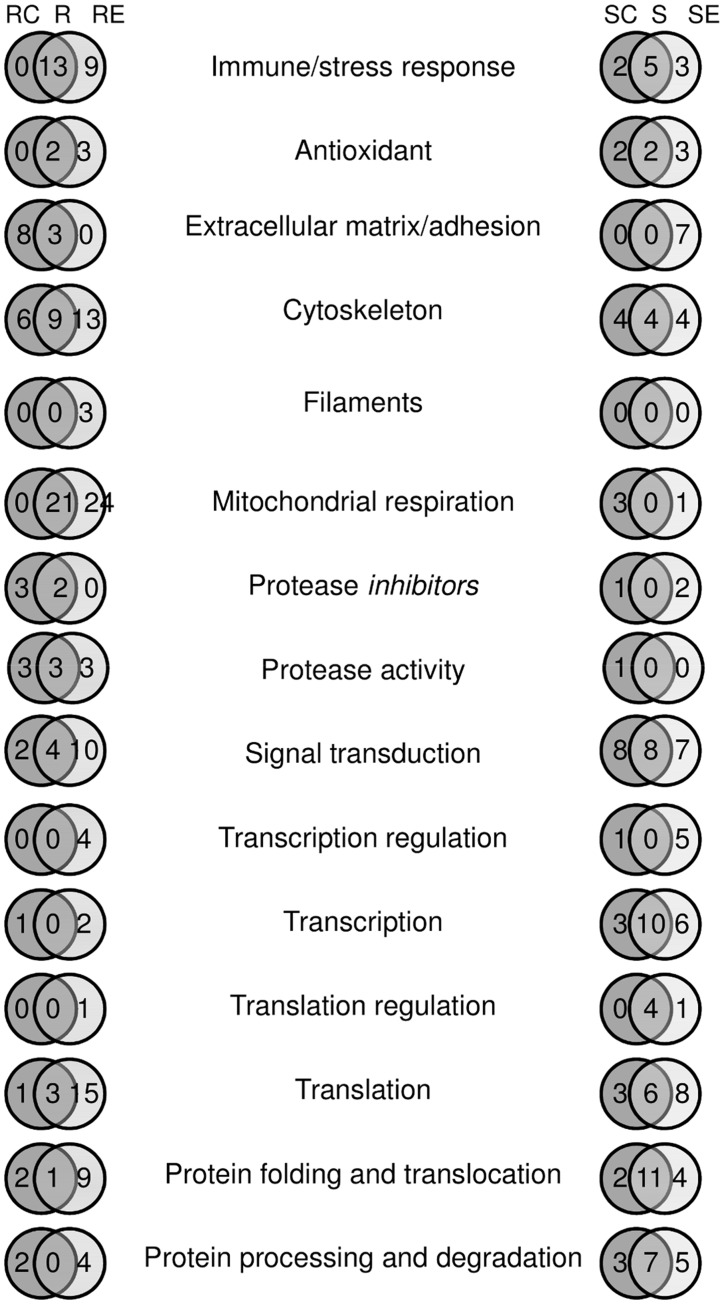
*B. glabrata* genes in different GO categories found to be differentially expressed. The number of differentially expressed genes in haemocytes from resistant (R) and susceptible (S) strains, before (C, control), both before and after (R/ S), and after *S. mansoni* exposure (E, exposed).

Genes found differently expressed between schistosome-resistant and schistosome-susceptible snails might be highly relevant for the interaction of those snails with *S. mansoni*. Differential levels of constitutive gene expression in haemocytes of the different strains *before* exposure may be responsible for the speedy response and elimination of *S. mansoni* in the resistant strain upon infection. In addition, differences between strains *after* exposure might give insight into the mechanism(s) of parasite elimination in resistant snails, since these are mounting a defence response; conversely, dissimilarities might indicate parasite interference with gene expression in susceptible snails. Therefore strain-specific differences should not be dismissed, although it is important to remember that other resistant and susceptible snail strains may show different responses. Gene homologues were identified for these differentially expressed transcripts and all were assigned GO annotations based on these homologies. The genes with identified GO categories were then classified into functional groups accordingly (File S2). The main functional groups represented cluster genes for known immune/stress response proteins, extracellular matrix/adhesion components, cytoskeletal proteins, mitochondrial respiratory chain proteins, signalling proteins, transcription and translation proteins and proteins that facilitate protein folding and degradation (Fig. 4). Immune/stress response genes with greater expression in resistant snail haemocytes included peptidoglycan recognition protein 1, FREPs 1 and 2, gram-negative binding protein, allograft inflammatory factor 1, heat shock protein (HSP) 40, ferritin, and glutathione-s-transferases (GSTs), all of which were differentially expressed irrespective of exposure, while FREPs 3 and 12, HSPs 70 and 90 showed greater expression post infection (File S2). Interestingly, HSP 60 was expressed to a greater extent in haemocytes of unexposed susceptible snails, with genes for the antimicrobial peptides hydramacin and neuromacin differentially expressed both before and after infection. Extracellular matrix/adhesion genes such as matrilin, dermatopontin 2, VWA domain-containing proteins and fibrillin were differentially expressed in resistant snail haemocytes with EGF-like domain containing protein, agrin, and a tandem repeat galectin showing lower expression after schistosome exposure. Unlike susceptible snails, a large number (45) of genes involved in mitochondrial respiration showed greater expression in haemocytes of resistant snails, with 24 of these showing greater expression post infection (Fig. 4). These genes included cytochrome b, cytochrome C oxidase subunits I-III, NADH dehydrogenase subunits 1, 3, 4 and 5, and ATPase subunits (File S2). In terms of signal transduction, differences were more balanced between the two strains with 16 genes showing greater expression in resistant snail haemocytes across all exposure regimes, as opposed to 23 genes in the susceptible (Fig. 4). Notable genes in the resistant phenotype included the protein tyrosine kinase src and protein kinase D, with 14-3-3 protein, G-protein coupled receptor kinase 2, twitchin, titin and nuclear factor κB (NFκB) inhibitor differentially expressed only following infection (File S2). In susceptible snail haemocytes, genes for rho GTPase activating protein, IKAP, and phosphoglycerate kinase were differentially expressed, with a dual specificity kinase and transforming growth factor β (TGFβ) receptor 1 involved only after infection (File S2). In terms of genes for cytoskeletal proteins, there was a preponderance of molecules involved in actin-related processes in haemocytes of the resistant strain compared to tubulin-related molecules in the susceptible strain (File S2); this situation persisted post-infection. Finally, encompassing all GO categories, genes that displayed the largest differences in expression (3.5-fold or greater) between snails included FREP2, Gram-negative bacteria binding protein, GSTσ, cytochrome b, NADH dehrogenase subunits 1 and 4, elastase 2, cystatin b, and endo 1,4 β glucanase in resistant snail haemoctyes, and polyprotein, endonuclease G, endonuclease mitochondrial precursor, and ATP-synthase-like protein, in susceptible snail haemocytes. HSP 90, and type 2 cystatin and fibropellin differed to such a degree only after infection for resistant and susceptible snail haemocytes, respectively (File S2).

### Gene Set Enrichment Analysis

**Figure 5:**
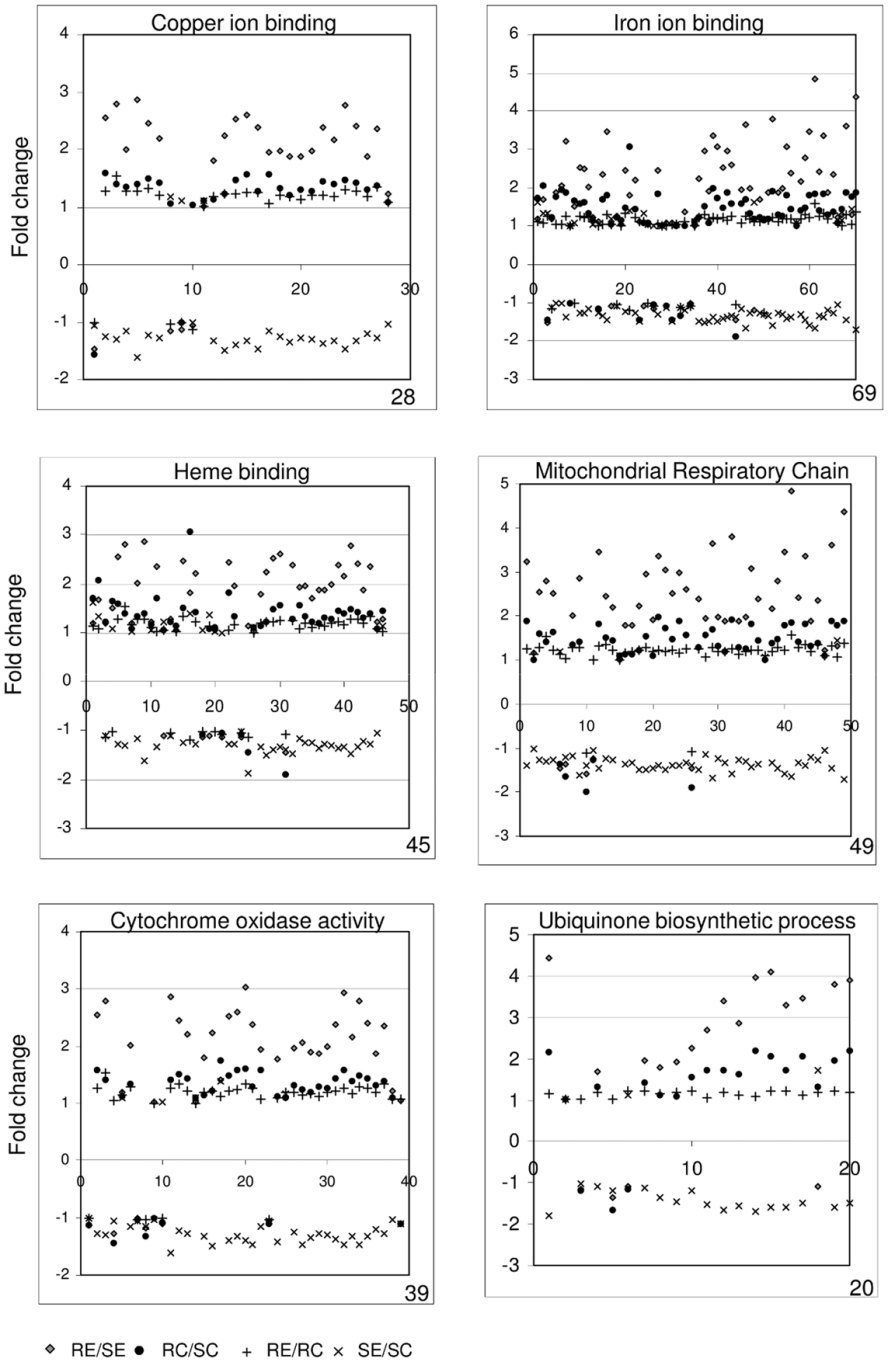
Gene set enrichment analysis (GSEA). The collated fold changes for each comparison (RE/SE, RC/SC, RE/RC, SE/SC) is shown for the genes in six selected GO categories. The order of the genes along the x-axis is arbitrary.

Gene set enrichment analysis (GSEA) [45], that identifies significant changes in expression of gene *groups* based on their function, rather than single genes, was employed for REvSE, RCvSC, RCvRE, SCvSC. We identified groups of GO terms (represented in bold in Table 3), all of which had higher expression levels in exposed resistant snails compared to unexposed (up-regulated on exposure), higher in resistant control snails compared to susceptible (strain-specific), higher in resistant exposed than susceptible exposed, and less in exposed susceptible snails than controls, (down-regulated in susceptible on exposure). Not every gene in each GO category followed the same trend, but the GSEA tests whether a significant number (more than would be expected by chance) are differently expressed in a particular category. For example, Fig. 5 illustrates 6 examples of GO categories in which the associated genes in general show positive fold changes (>+1) for REvSE, RCvSC and REvRC, and negative fold changes (>**−**1) for SEvSC. This indicates that even between control and unexposed snails, expression in R was greater than S, and that a significant number of the associated genes are up-regulated post-exposure in the schistosome-resistant snails, but down-regulated in the schistosome-susceptible snails; there is also, therefore, an even larger difference in expression of these genes when comparing RE with SE, with resistant being greater than susceptible. The gene groups identified as following these trends are primarily involved in mitochondrial respiratory process and ubiquinone biosynthetic processes, both indicative of increased metabolic activity consistent with mounting a defence response.

**Table 3:** Gene Set Enrichment Analysis (GSEA).

Upregulation (AvB A>B)							
GO ID	Gene Ontology	Type of GO	No.genes	REvSE	RCvSC	REvRC	SEvSC
GO:0009060	**aerobic respiration**	biological_process	21	**2.78E−12**	**4.31E−09**	**1.88E E−09**	1
GO:0005507	**copper ion binding**	molecular_function	28	**2.47E E−11**	**3.33E E−08**	**9.73E E−10**	1
GO:0004129	**cytochrome-c oxidase activity**	molecular_function	39	**3.91E E−12**	**4.96E E−07**	**7.38E E−11**	1
GO:0009055	electron carrier activity	molecular_function	47	**1.85E E−06**	**4.19E E−08**	**2.85E E−05**	0.996695
GO:0006118	**electron transport**	biological_process	106	**3.97E E−12**	**8.96E E−11**	**8.43E E−12**	1
GO:0020037	**heme binding**	molecular_function	45	**1.56E E−13**	**1.30E E−10**	**8.37E E−07**	0.99999
GO:0016021	**integral to membrane**	cellular_component	126	**2.74E E−10**	**7.52E E−11**	**9.89E E−12**	1
GO:0005506	**iron ion binding**	molecular_function	69	**0**	**2.15E E−14**	**3.33E E−12**	1
GO:0006123	**mitochondrial electron transport, cytochrome c to oxygen**	biological_process	39	**3.91E E−12**	**4.96E E−07**	**7.38E E−11**	1
GO:0006120	**mitochondrial electron transport, NADH to ubiquinone**	biological_process	23	**3.43E E−05**	0.001246	**4.49E E−06**	0.999995
GO:0005746	**mitochondrial respiratory chain**	cellular_component	49	**0**	**1.16E E−12**	**0**	1
GO:0008137	**NADH dehydrogenase (ubiquinone) activity**	molecular_function	19	**9.68E E−08**	**1.41E E−05**	**2.55E E−06**	1
GO:0015992	**proton transport**	biological_process	73	**1.15E E−12**	**8.49E E−07**	**8.75E E−12**	1
GO:0045277	**respiratory chain complex IV**	cellular_component	36	**2.46E E−12**	**2.73E E−07**	**5.24E E−11**	1
GO:0022904	respiratory electron transport chain	biological_process	7	**6.79E E−05**	0.000196	0.000403	0.995547
GO:0006814	**sodium ion transport**	biological_process	24	**3.16E E−07**	**5.69E E−05**	**0.000109**	0.999999
GO:0006810	transport	biological_process	58	0.0003	**7.62E E−06**	**3.29E E−05**	0.953241
GO:0006744	**ubiquinone biosynthetic process**	biological_process	20	**3.49E E−07**	**7.20E E−06**	**1.02E E−06**	0.999997
Downregulation (AvB A<B)							
GO ID	Gene Ontology	Type of GO	No.genes	REvSE	RCvSC	REvRC	SEvSC
GO:0009060	**aerobic respiration**	biological_process	21	1	1	1	**3.72E E−07**
GO:0005507	**copper ion binding**	molecular_function	28	1	1	1	**5.21E E−09**
GO:0005524	ATP binding	molecular_function	154	0.66052	0.04391	0.334357	**2.19E E−06**
GO:0004129	**cytochrome-c oxidase activity**	molecular_function	39	1	1	1	**9.28E E−10**
GO:0006118	**electron transport**	biological_process	106	1	1	1	**3.10E E−09**
GO:0020037	**heme binding**	molecular_function	45	1	1	0.999999	**9.86E E−06**
GO:0016021	**integral to membrane**	cellular_component	126	1	1	1	**2.11E E−07**
GO:0005506	**iron ion binding**	molecular_function	69	1	1	1	**1.40E E−09**
GO:0006123	**mitochondrial electron transport, cytochrome c to oxygen**	biological_process	39	1	1	1	**9.28E E−10**
GO:0006120	**mitochondrial electron transport, NADH to ubiquinone**	biological_process	23	0.99997	0.998755	0.999996	**4.82E E−06**
GO:0005746	**mitochondrial respiratory chain**	cellular_component	49	1	1	1	**0**
GO:0005739	mitochondrion	cellular_component	48	0.93988	0.800927	0.817296	**8.18E E−06**
GO:0008137	**NADH dehydrogenase (ubiquinone) activity**	molecular_function	19	1	0.999986	0.999997	**2.42E E−07**
GO:0015992	**proton transport**	biological_process	73	1	0.999999	1	**5.85E E−14**
GO:0045277	**respiratory chain complex IV**	cellular_component	36	1	1	1	**9.42E E−11**
GO:0006814	**sodium ion transport**	biological_process	24	1	0.999943	0.999891	**8.06E E−07**
GO:0006744	**ubiquinone biosynthetic process**	biological_process	20	1	0.999993	0.999999	**2.96E E−06**
GO:0005840	ribosome	cellular_component	85	0.03859	**1.20E E−05**	0.723301	0.000821
GO:0006457	protein folding	biological_process	21	0.00041	**3.17E E−06**	0.55687	0.00087
GO:0006094	gluconeogenesis	biological_process	22	0.01078	**3.09E E−05**	0.163101	0.002104
GO:0005634	nucleus	cellular_component	93	0.00057	**7.58E E−08**	0.533302	0.00296
GO:0006096	glycolysis	biological_process	20	0.00633	**2.13E E−05**	0.220296	0.003285
GO:0003735	structural constituent of ribosome	molecular_function	105	0.05398	**6.79E E−06**	0.970198	0.021822
GO:0006412	translation	biological_process	98	0.04483	**6.10E E−05**	0.88094	0.028665
GO:0042254	ribosome biogenesis	biological_process	108	0.04124	**2.39E E−06**	0.989179	0.03393
GO:0005615	extracellular space	cellular_component	26	0.00024	**0.000126**	0.019181	0.044914
GO:0005515	protein binding	molecular_function	240	0.00035	**3.62E E−06**	0.836396	0.33465
GO:0003743	translation initiation factor activity	molecular_function	9	**5.79E E−05**	**8.26E E−06**	0.278141	0.050926
GO:0006446	regulation of translational initiation	biological_process	8	**9.10E E−05**	**1.99E E−05**	0.249216	0.074297
GO:0051082	unfolded protein binding	molecular_function	27	**0.00013**	**2.34E E−05**	0.878669	0.135328
GO:0003924	GTPase activity	molecular_function	67	**9.99E E−05**	**9.11E E−05**	0.262811	0.315466
GO:0007018	microtubule-based movement	biological_process	47	**9.39E E−06**	**3.30E E−05**	0.422782	0.744616
GO:0005874	microtubule	cellular_component	59	**6.63E E−06**	**1.04E E−05**	0.440339	0.745536
GO:0003723	RNA binding	molecular_function	55	**1.00E E−05**	**1.02E E−05**	0.730866	0.809142
GO:0051258	protein polymerization	biological_process	32	**3.37E E−06**	0.000186	0.848843	0.988085

The gene ontologies (GO) listed were significantly different (adjusted p <0.01) in at least one of the 4 comparisons. Significant p values (Bonferri adjusted) are shown in **bold**. GOs that were found to be up-regulated in resistant snails **and** down-regulated in susceptible are also shown in **bold**.

### FatiScan Analysis to Identify Trends in Gene Expression

**Figure 6:**
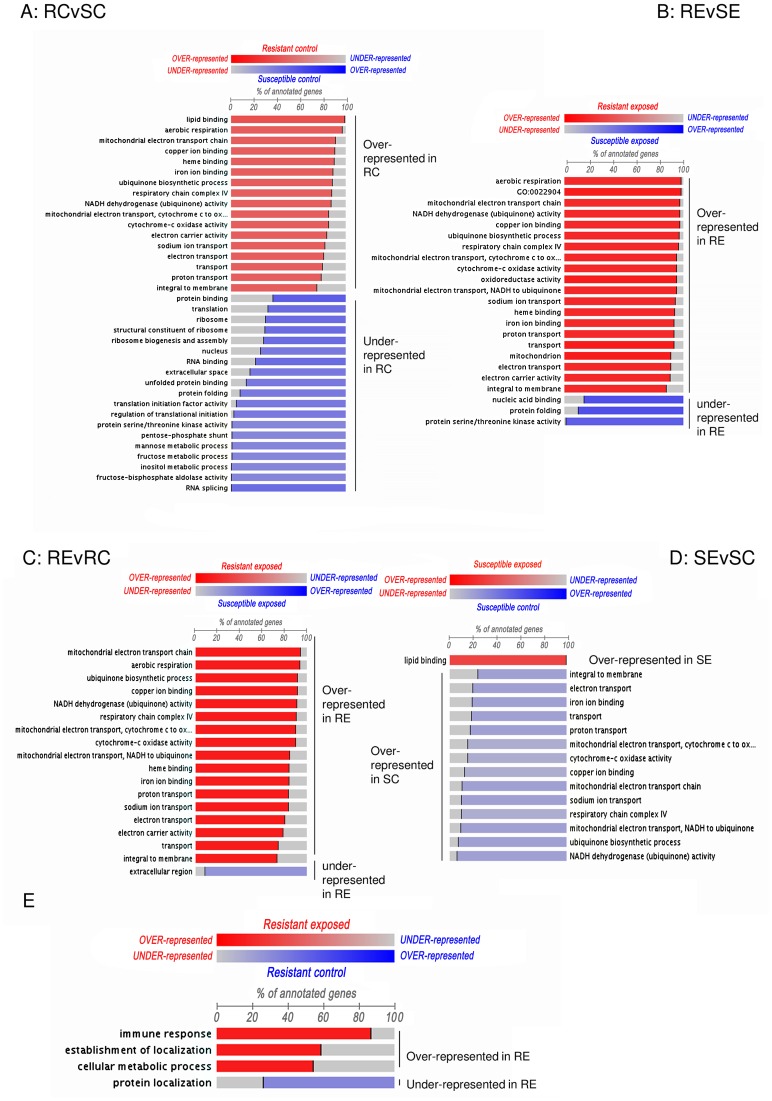
FatiScan analysis illustrating gene expression fold change ranked lists from *B. glabrata* microarray comparisons using custom array annotation. A. Resistant control (RC) compared to susceptible control (SC). B. Resistant exposed (RE) compared to susceptible exposed (SE). C. Resistant exposed (RE) to resistant control (RC). D. Susceptible exposed (SE) compared to susceptible control (SC). E. RE compared to RC using level 3 GO annotations. Significantly differentially represented GO categories are listed for each comparison. Bar leading from the left, with GO category label on the left indicates *over* representation in the upper category (shown above each diagram), label on the right means *under* represented. Bar leading from the right and label on the right indicates *over*-representation in the lower category and label on the left *under-*represented.

FatiScan analysis [46] was used to detect asymmetrical distribution of GO categories from the fold change ranked list for each of the 4 haemocyte comparisons (RCvSC, REvSE, REvRC, and SEvSC). Distinct differences were identified between the snail strains both before and after infection, and the types of genes found to differ between haemocytes confirm the GSEA results (Fig. 6 A–B). Most striking, however, were the differences *in response* to the schistosome shown by the snail strains. Haemocytes of resistant snails exposed to *S. mansoni* showed an over-representation of haemocyte genes involved in mitochondrial respiratory processes and ubiquinone biosynthetic processes (Fig. 6C), demonstrating these are up-regulated in response to the parasite (as well as being already elevated in control resistant snails compared to susceptible), while in haemocytes of the susceptible snails these same genes were under-represented (Fig. 6D) indicating that their expression was suppressed by the parasite. This is also consistent with the findings of the GSEA analysis. FatiScan analysis of level 3 GO annotations also identified that the resistant exposed snails switched on immune response genes upon exposure to *S. mansoni* (Fig. 6E), again showing an active response to infection.

## Discussion

The genes differentially expressed between haemocytes of the schistosome-susceptible and schistosome-resistant *B. glabrata* strains offer great insight into the complex molecular processes that are involved in the defence response to the parasite. Fifty-nine of the 98 genes that we identified in our initial investigation of strain differences using the considerably smaller (2****K versus 5****K) previous array platform [36] have been confirmed, and added to, by the current experiment. Again, we observed differential expression (elevated in resistant snails) of genes involved in energy metabolism, and transcription and translation indicating a general increase in cellular activity, consistent with generating the necessary components for mounting a defence response. Perhaps of greater interest is the finding of a considerably different response in susceptible and resistant snail strains as early as 2 hr post exposure to *S. mansoni*.

Stress response genes were identified in the set of genes that were present in the resistant snails both before and after exposure, as well as in the exposed resistant snails. FREPs, a unique family of molluscan calcium-dependent lectins, are known to be up-regulated following parasite infection and to bind to parasite surfaces [9], likely through interaction with parasite mucins [21], [22]. Knockdown of FREP3 in *B. glabrata* resistant to *E. paraensei* by RNA interference (RNAi) resulted in a phenotype switch, whereby 31% of RNAi-treated snails became susceptible to the parasite [11]. Suppression of FREP3 in *B. glabrata* resistant to *S. mansoni* has also been shown to increase susceptibility to this schistosome with 20% of snails becoming infected [20]. Thus FREP3 seems to play some role in defence against *S. mansoni* concordant with the view that the primary function of fibrinogen-domain containing proteins in invertebrates is in protection against infection, rather than coagulation [62]. Here, we have demonstrated that FREP3 and FREP12 expression was greater in haemocytes of resistant snails compared to susceptible snails post miracidial exposure, whereas FREPS 1 and 2 were differentially expressed irrespective of exposure. Thus it seems possible that in the *B. glabrata*/*S. mansoni* infection model, FREP3 might represent a molecule vital to the maintenance of the resistance phenotype and that high levels of FREP expression in general might facilitate early parasite recognition.

Expression of a HSP 70 gene was also greater in resistant snail haemocytes after schistosome infection confirming of our previous findings [36], [42], but this contrasts with the findings of Ittiprasert *et al* (2009), who demonstrated up-regulation of HSP70 in susceptible juvenile snails but not resistant [63]. Temperature stresses have also recently been suggested to affect susceptibility of snails to schistosome infection in conjunction with changes to expression levels of HSP transcripts [64]. Interestingly Zahoor *et al* (2010) demonstrated that *S. mansoni* ESPs, derived from larvae transforming from miracidia to mother sporocysts, reduced the quantity of HSP 70 protein in haemocytes of both snail strains 1 h after exposure to ESPs and that HSP 70 protein levels were also lower 35 days after infection [48]. Given that this molecule has important intracellular chaperone and extracellular immunomodulatory capacities [65], it would be valuable to elucidate the temporal dynamics of schistosome infection on HSP 70 gene and protein expression in detail together with that for the three other differentially expressed HSP genes, in order to fully understand the role they may play in snail responses to schistosome infection.

Differential expression between haemocytes of resistant and susceptible control snails, of matrillin, dermatopontin and other transcripts involved in cell adhesion may also have a significant bearing on host-parasite interactions. Bouchut *et al* (2006) investigated gene expression of several cell adhesion genes in *B. glabrata* strains resistant or susceptible to *E. caproni* and found dermatopontin 2 and matrillin to be differentially expressed [66]; however, in their snail strains matrillin was over expressed in snails susceptible to a different parasite, *E. caproni*, and they hypothesized that susceptible snails may possess a more potent haemolymph coagulation system preventing or slowing haemocyte migration [66]. Here we identified six of the genes investigated by these authors as being significantly present in resistant control snails and three (two the same) that were different between haemocytes of resistant snails and susceptible snails both before and after exposure. Although different expression patterns were found in this study, it may emphasize the importance of these genes in snail-parasite interactions, although their roles are not currently clear. That fewer differentially expressed genes were found after exposure may suggest that the susceptible snails up-regulated these genes, whereas the resistant snails already expressed these genes, even in control snails. A gene for tandem repeat galectin showed greater expression in susceptible snail haemocytes after *S. mansoni* infection. This finding is curious given that these molecules are expressed on the surface of ∼60% of *B. glabrata* haemocytes, that they bind the *S. mansoni* sporocyst tegument *via* interaction with surface-exposed LacNAc sugars, and that susceptible snail haemocytes do not encapsulate *S. mansoni* miracidia/sporocysts [33]. However, if developing larvae release sufficient LacNAc into the haemolymph during transformation, binding to the haemocyte receptors could serve to dampen recognition of and/or responses to the parasite either directly, or through interference of haemocyte signalling mechanisms as demonstrated for other sugar molecules [67], [68]. Cell surface receptors such as integrins bind to extracellular matrix components and facilitate cell migration through tissues towards invading pathogens. These events are communicated intracellularly and cell movements, including encapsulation and phagocytosis, are then facilitated by actin and a variety of actin binding proteins. In this context it is notable that resistant snail haemocytes displayed a preponderance of genes involved in actin-related processes either in the presence or absence of schistosome infection. This implies that these haemocytes might display enhanced phagocytic and migratory capacity when compared to their susceptible snail counterparts benefiting the anti-parasite response.

The GSEA and FatiScan analyses also highlight that genes involved in mitochondrial respiration and ubiquinone degradation were already active in haemocytes of the resistant snails prior to schistosome exposure and that in these snails they were further activated, while in the susceptible snails they were suppressed, demonstrating a significant difference in the response of resistant and susceptible snails to schistosome exposure. Genes required for copper, iron and heme-binding were also differentially expressed, although they may function in energy production too; for example, haemoglobins that possess a high oxygen affinity are present as blood respiratory proteins in *B. glabrata* [69]. Hanelt *et al* (2008) also found indications of up-regulation of heme and metal ion-binding in response to bacterial and *S. mansoni* challenge (12****hr post-exposure) [14]. Ferritin, also identified in resistant snails in earlier gene expression studies [44] and recently identified in an RNA-seq based approach to identify immune responses following bacterial or yeast challenges in *B. glabrata* [70] stores iron in a non-toxic form, enabling deposition of iron in a safe form and transport to areas where iron is required. Ferrous iron (Fe^2+^) is toxic to cells as it acts as a catalyst in the formation of the hydroxyl free radical (OH•) from hydrogen peroxide (H_2_O_2_). Given the importance of these reactive oxygen species to killing of *S. mansoni* sporocysts [8], [28], [71], greater ferritin expression could be relevant in terms of its capacity to affect the cellular balance of H_2_O_2_ and OH• possibly influencing the outcome of infection. In the current study, resistant snail haemocytes were found to also differentially express GSTs that would serve as antioxidants to prevent cellular damage to the haemocytes. In the context of energy production, genes involved in respiratory chain and ATP production have been shown to differ between two oyster species that vary in their response to the parasite *Perkinsus marinus* [72]. The above suggests that the resistant snails were already expressing many of the genes required for defence responses prior to exposure.

This study provides the first evidence from global gene expression analyses that not only is there is a fundamental difference in the defence physiology between the snail strains used here before infection, but that the resistant snails actively respond to the schistosome, while the susceptible snails react in an opposing fashion by suppressing expression of the types of genes which are activated in a responding (resistant) snail, consistent with the notion that the schistosome is producing molecules that interfere with the snail’s defence response [68]. Hanington *et al* (2010) found that an initial (0.5–2 day post infection) up-regulation of immune/stress response genes in susceptible *B. glabrata* was followed by a stronger down-regulation later during infection with *S. mansoni*, that was in contrast to *E. paraensei* exposed snails which showed down-regulation from 0.5 day post-infection [38]. They concluded that both parasites were able to interfere with host defense responses, but that *E. paraensei* was able to do this more rapidly and robustly than *S. mansoni*. Our results also suggest interference by the parasite, but in contrast to Hanington *et al* (2010) [38], we show that this phenomenon is occurring only 2 hours after exposure. Such early inactivation is coincident with early post-embryonic development of the parasitic sporocyst larval stage, a crucial phase when the schistosome lays down a new tegument and is perhaps more exposed to the host immune system while the snail is exposed to ESPs and ciliated plates released from schistosomes during their development [73].

In conclusion, this microarray experiment, by determining the expression of a large number of genes simultaneously, many more than can be investigated by qPCR, has enabled the construction of a framework of processes involved in haemocyte responses during the first phase of schistosome infection. The resistant snails, even before infection, express many different genes compared with susceptible snails and in many respects seem to be primed and ready to respond to schistosome attack. The resistant snails also demonstrated activation of defence processes, while the susceptible snails displayed inactivation. Pinpointing individual genes significantly affected by parasite exposure may have been made more difficult by biological variation both in schistosome penetration time (after addition of miracidia to snail water) and in individual snail responses since to obtain sufficient material it was necessary to pool haemocytes. Alternatively, the actual gene expression changes at this early stage after schistosome exposure, may be subtle and therefore difficult to detect resulting in a skewed outcome whereby strain-specific differences in gene expression outweighed parasite-induced changes. Nevertheless, by integrating GSEA and FatiScan analysis, the outcomes detailed in this paper have enabled a holistic view of changes in gene expression as a consequence of phenotype and exposure regime. Statistical analysis of clustered gene expression within any particular category has provided enhanced confidence in the relative importance of changes that might result in altered cellular physiology. In this way, this study provides a new way of assessing the complex biology of snail-schistosome interactions giving insight that will help future studies to identify mechanisms of compatibility in this fascinating host-parasite system.

## Supporting Information

File S1 **Annotation file for the genes represented on the 5 K** ***B. glabrata*** **microarray.**
(TXT)

File S2 **Genes identified as significantly differentially expressed between resistant and susceptible** ***B. glabrata*** **snails, before exposure (C-control), after exposure to** ***S. mansoni*** **(E-exposed) and both before and after (not affected by exposure).** Genes with no known homologues are not shown. fc- fold change, figure in grey = no significant difference. *Genes *previously* identified as being significantly different between schistosome-resistant and -susceptible strains of *B. glabrata* [36]
(DOC)
