# Supplementary material for: Early Differential Gene Expression in Haemocytes from Resistant and Susceptible Biomphalaria glabrata Strains in Response to Schistosoma mansoni
Source: PLoS One. 2012 Dec 26;7(12):e51102. doi: 10.1371/journal.pone.0051102 (PMC3530592; doi:10.1371/journal.pone.0051102)
Supplement: File S2 — Genes identified as significantly differentially expressed between resistant and susceptible B. glabrata snails, before exposure (C-control), after exposure to S. mansoni (E-exposed) and both before and after (not affected by exposure). Genes with no known homologues are not shown. fc- fold change, figure in grey = no significant difference. *Genes previously identified as being significantly different between schistosome-resistant and -susceptible strains of B. glabrata [36] (DOC) [file pone.0051102.s002.doc]

| Acc No. | Sequence desc. | bp | Hit ACC | E-Value | fc (RC/SC) | fc (RE/SE) |
| --- | --- | --- | --- | --- | --- | --- |
| Resistant Control only | |  |  |  |  |  |
| Extracellular matrix/adhesion | |  |  |  |  |  |
| CK656770.1 | Matrilin | 205 | AAN61407 | 1.57E-05 | 1.54 | 1.22 |
| CN476089.1 | Matrilin | 527 | AAN61407 | 3.68E-49 | 1.50 | 1.08 |
| DW474806.1 | collagen alpha-5 chain precursor | 558 | BAD16597 | 1.82E-09 | 2.34 | -1.40 |
| CK989678.1 | Bg vwa domain containing protein 2 | 638 | AAZ80792 | 5.50E-42 | 1.66 | 1.34 |
| CK989671.1 | Bg vwa domain-containing protein 3 | 569 | AAZ80793 | 4.71E-46 | 2.02 | 1.67 |
| CK988741.1 | dermatopontin 2 | 552 | AAZ80785 | 4.61E-19 | 3.01 | 1.89 |
| CO870431.1 | Fibrillin | 730 | XP_002207842 | 9.77E-18 | 1.41 | 1.03 |
| CV548577.1 | multiple EGF and TSP domain-containing protein | 691 | AAZ80797 | 8.03E-80 | 1.67 | 1.13 |
|  |  |  |  |  |  |  |
| Cytoskeleton |  |  |  |  |  |  |
| CK989030.1 | beta-thymosin domain repeat protein csp29kda_v1 | 560 | AAN08022 | 1.10E-07 | 2.00 | 1.51 |
| CK149163.1 | tropomyosin | 620 | P43689 | 2.42E-39 | 1.78 | 1.24 |
| CK656807.1 | tropomyosin | 607 | P43689 | 5.26E-52 | 1.80 | 1.17 |
| CK988934.1 | tropomyosin | 609 | P43689 | 4.51E-29 | 1.58 | 1.18 |
| EW996906.1 | thymosin isoform 2 | 657 | AAN08022 | 1.06E-30 | 1.99 | 1.55 |
| CO870300.1 | Non muscle myosin II | 806 | AAZ40189 | 9.50E-84 | 1.44 | 1.15 |
|  |  |  |  |  |  |  |
| Signal transducers | |  |  |  |  |  |
| CK149232.1 | src-family protein tyrosine kinase | 615 | ACI23622 | 1.26E-40 | 1.88 | 1.63 |
| EW996690.1 | gtpase activating protein (sh3 domain) binding protein 2 | 687 | XP_002203534 | 3.63E-24 | 1.49 | 1.33 |
|  |  |  |  |  |  |  |
| Transcription |  |  |  |  |  |  |
| CN013314.1 | CCR4-NOT transcription complex, subunit 2 | 320 | XP_002074671 | 6.01E-21 | 1.54 | 1.39 |
|  |  |  |  |  |  |  |
| Translation |  |  |  |  |  |  |
| EW997450.1 | large subunit ribosomal protein 6 | 426 | XP_321154 | 9.20E-14 | 1.98 | 1.63 |
|  |  |  |  |  |  |  |
| Protein trafficking and translocation | |  |  |  |  |  |
| CK656883.1 | snare protein | 190 | YP_834194 | 9.56E-05 | 1.35 | 1.13 |
| CK656836.1 | solute carrier family 2 (facilitated glucose transporter) member 13 | 714 | XP_002220289 | 5.85E-60 | 1.43 | 1.31 |
|  |  |  |  |  |  |  |
| Protein processing and degredation | |  |  |  |  |  |
| CK989562.1 | apg3 autophagy 3-like | 450 | XP_002224846 | 1.32E-44 | 1.59 | 1.38 |
| CV548704.1 | palmitoyl-protein thioesterase 1 | 559 | XP_001631725 | 7.49E-73 | 1.40 | 1.08 |
|  |  |  |  |  |  |  |
| Serine protease inhibitors | |  |  |  |  |  |
| DW474282.1 | proteinase inhibitor i4 serpin | 649 | ZP_06248962 | 2.92E-08 | 1.94 | 1.08 |
| DW474671.1 | serine (or cysteine) proteinase inhibitor clade b member 3 | 574 | XP_001141918 | 1.30E-13 | 3.39 | -1.30 |
| CK640646.1 | leukocyte elastase inhibitor-like | 284 | XP_002736674 | 8.13E-04 | 2.53 | 1.10 |
|  |  |  |  |  |  |  |
| Proteases |  |  |  |  |  |  |
| CN549149.1 | cathepsin l | 543 | CAI43320 | 1.83E-08 | 2.10 | -1.11 |
| CK989294.1 | astacin family metalloendopeptidase farm-1 | 500 | XP_002236281 | 9.35E-14 | 2.04 | 1.57 |
| CK989935.1 | astacin-like protein | 603 | AAX56337 | 1.38E-04 | 1.54 | 1.21 |
|  |  |  |  |  |  |  |
| Cell division |  |  |  |  |  |  |
| CV548384.1 | Lin-5 family member protein | 655 | XP_001019700 | 6.85E-06 | 1.57 | 1.18 |
|  |  |  |  |  |  |  |
| Ig containing |  |  |  |  |  |  |
| CV548156.1 | neural ectodermal development factor imp-l2 | 708 | XP_002411991 | 1.02E-24 | 1.92 | 1.26 |
|  |  |  |  |  |  |  |
| Iron homeostasis | |  |  |  |  |  |
| CV548600.1 | ferric-chelate reductase 1 | 281 | NP_001077164 | 3.45E-05 | 1.72 | 1.29 |
|  |  |  |  |  |  |  |
| Misc |  |  |  |  |  |  |
| CO870241.1 | mannose c type 1 | 691 | CAF97168 | 5.86E-14 | 1.67 | 1.22 |
| CK989348.1 | serine acetyltransferase | 495 | ABM53545 | 2.39E-30 | 1.68 | 1.29 |
| EW996861.1 | slc3a2 protein | 469 | NP_001080246 | 2.97E-12 | 1.46 | 1.24 |
| CV548146.1 | Selenoprotein W2b | 631 | AAX61159 | 5.53E-10 | 1.41 | 1.32 |
| EW996695.1 | fumarylacetoacetate hydrolase domain containing 1 | 573 | XP_001640847 | 1.16E-39 | 1.37 | 1.05 |
|  |  |  |  |  |  |  |
| Resitant control and exposed | |  |  |  |  |  |
| Acc No | Sequence desc. | bp | Hit ACC | E-Value | fc (RC/SC) | fc (RE/SE) |
|  |  |  |  |  |  |  |
| Immune/stress response/innate immunity | |  |  |  |  |  |
| CK989530.1 | peptidoglycan recognition protein l | 177 | BAH66799 | 3.45E-05 | 1.90 | 1.49 |
| EW997506.1 | BgMFREP1 | 314 | AAK13549 | 3.53E-37 | 1.67 | 1.68 |
| CK989205.1 | allograft inflammatory factor 1 | 633 | ACJ65689 | 9.30E-42 | 2.20 | 1.83 |
| EW997242.1 | FREP2 | 396 | AAC47700 | 8.98E-17 | 2.16 | 2.53 |
| CK989005.1 | FREP2 | 507 | AAC47700 | 1.27E-08 | 2.14 | 2.35 |
| AY012700.1 | FREP2 | 6072 | AAC47700 | 6.14E-127 | 2.33 | 3.84 |
| CO870354.1 | ferritin | 225 | AAS66655 | 5.28E-09 | 2.13 | 1.91 |
| CK989447.1 | ferritin | 379 | P42577 | 2.78E-20 | 1.49 | 1.40 |
| CK988667.1 | gram-negative bacteria binding protein | 397 | ABO40828 | 2.26E-60 | 3.84 | 2.11 |
| CV548196.1 | HSP40 | 441 | NP_758841 | 5.45E-14 | 1.83 | 1.43 |
| CK988734.1 | glutathione s-transferase, sigma class | 592 | ABO26604 | 1.62E-16 | 6.93 | 4.64 |
| CK989663.1 | glutathione s-transferase, mu | 644 | ABS32298 | 2.39E-17 | 2.08 | 2.05 |
| CK989010.1 | glutathione s-transferase, mu | 696 | ACE79172 | 9.40E-44 | 1.84 | 2.02 |
|  |  |  |  |  |  |  |
| extracellular matrix/adhesion | |  |  |  |  |  |
| CK989715.1 | dermatopontin 3 | 588 | AAZ80787 | 1.72E-90 | 2.85 | 1.75 |
| CK988686.1 | dermatopontin 2 | 392 | AAZ80785 | 7.62E-37 | 3.46 | 1.92 |
| CK988725.1 | matrilin | 545 | AAZ80784 | 7.30E-36 | 1.60 | 1.57 |
|  |  |  |  |  |  |  |
| cytoskeleton |  |  |  |  |  |  |
| CV548376.1 | actin | 519 | P92179 | 4.72E-78 | 1.70 | 2.06 |
| CK988792.1 | actin monomer binding protein | 554 | XP_566890 | 1.81E-07 | 1.98 | 1.60 |
| CK989538.1 | actin, gamma 1 | 329 | BAE73006 | 1.85E-14 | 1.63 | 1.60 |
| CV548402.1 | beta-thymosin domain repeat protein csp24kda_v1 | 614 | AAN08024 | 3.01E-17 | 2.10 | 1.58 |
| CV548764.1 | beta-thymosin isoform 2 | 735 | AAN08024 | 1.59E-15 | 1.90 | 1.53 |
| EW997401.1 | myosin regulatory light chain smooth muscle isoform | 500 | CAH04894 | 3.93E-12 | 2.05 | 3.04 |
| EW997192.1 | myosin regulatory light chain smooth muscle isoform | 555 | CAH04894 | 1.46E-12 | 1.65 | 2.51 |
| CO870396.1 | tropomyosin | 781 | P43689 | 4.31E-78 | 2.02 | 1.54 |
| CK988897.1 | WD-repeat protein | 485 | XP_002429978 | 1.53E-04 | 1.69 | 1.62 |
|  |  |  |  |  |  |  |
| mitochondrial respiratory chain | |  |  |  |  |  |
| EW997595.1 | cytochrome b | 971 | AAQ74246 | 1.52E-151 | 1.89 | 3.62 |
| EW997470.1 | cytochrome b | 804 | AAQ74246 | 7.47E-109 | 1.83 | 3.36 |
| EW997361.1 | cytochrome b | 810 | AAQ74246 | 1.99E-122 | 1.85 | 4.83 |
| EW997320.1 | cytochrome b | 483 | AAQ74246 | 3.14E-70 | 1.79 | 3.47 |
| EW997240.1 | cytochrome b | 757 | AAQ74246 | 9.76E-112 | 1.81 | 3.08 |
| EW997136.1 | cytochrome b | 935 | AAQ74246 | 1.17E-150 | 1.93 | 3.80 |
| EW996899.1 | cytochrome b | 645 | AAQ74246 | 2.53E-98 | 1.88 | 2.98 |
| EW996844.1 | cytochrome b | 890 | AAQ74246 | 3.29E-99 | 1.99 | 3.37 |
| EW996799.1 | cytochrome b | 797 | AAQ74246 | 1.06E-95 | 1.53 | 2.96 |
| CN549147.1 | cytochrome b | 607 | AAQ74246 | 1.42E-65 | 1.82 | 3.46 |
| CK989123.1 | cytochrome b | 517 | NP_976168 | 4.79E-46 | 1.64 | 2.51 |
| CK656796.1 | cytochrome b | 339 | NP_976168 | 1.43E-35 | 1.87 | 3.23 |
| EW996873.1 | cytochrome c oxidase subunit i | 802 | YP_001739865 | 1.47E-63 | 1.59 | 2.89 |
| EW996798.1 | cytochrome oxidase subunit i | 575 | AAK52610 | 6.36E-46 | 1.47 | 2.28 |
| EW997619.1 | nadh dehydrogenase subunit 1 | 501 | NP_976166 | 6.16E-45 | 1.95 | 3.80 |
| EW997655.1 | nadh dehydrogenase subunit 4 | 579 | NP_976173 | 8.46E-56 | 2.19 | 3.89 |
| EW997387.1 | nadh dehydrogenase subunit 4 | 508 | NP_976173 | 3.78E-53 | 2.06 | 3.46 |
| EW997322.1 | nadh dehydrogenase subunit 4 | 466 | NP_976173 | 9.80E-48 | 2.06 | 4.08 |
| EW997275.1 | nadh dehydrogenase subunit 4 | 629 | NP_976173 | 6.14E-62 | 2.18 | 3.97 |
| CK989056.1 | nadh dehydrogenase subunit 4 | 516 | NP_976173 | 3.47E-25 | 1.77 | 2.99 |
| CK149168.1 | nadh dehydrogenase subunit 4 | 317 | NP_976173 | 2.98E-36 | 2.16 | 4.42 |
|  |  |  |  |  |  |  |
| Signal transducers | |  |  |  |  |  |
| CK989297.1 | EGF domain containing protein | 438 | XP_002122318 | 8.48E-04 | 1.76 | 1.73 |
| CN549178.1 | src-family protein tyrosine kinase | 442 | ACI23622 | 3.09E-17 | 1.73 | 1.61 |
| EW997524.1 | transmembrane serine/threonine protein kinase D | 487 | ACD54679 | 1.74E-12 | 1.93 | 2.79 |
| CK989292.1 | stanniocalcin | 528 | ABY87355 | 3.20E-24 | 1.66 | 1.45 |
|  |  |  |  |  |  |  |
| Transcription regulator | |  |  |  |  |  |
| CO870341.1 | cytochrome p450 | 343 | P48416 | 3.41E-32 | 3.06 | 1.82 |
|  |  |  |  |  |  |  |
| Translation (GO:0006412) | |  |  |  |  |  |
| EW997536.1 | ribosomal protein s20 | 417 | CAL69110 | 3.91E-20 | 2.03 | 2.25 |
| EW997381.1 | ribosomal protein rpl13a | 388 | ABW23219 | 2.22E-14 | 2.05 | 2.69 |
| EW996828.1 | ribosomal protein s13 | 574 | AAX43326 | 1.56E-68 | 1.46 | 1.88 |
|  |  |  |  |  |  |  |
| Protein trafficking | |  |  |  |  |  |
| CO870346.1 | islet cell autoantigen 1 | 604 | ACN11438 | 7.82E-16 | 2.26 | 3.26 |
|  |  |  |  |  |  |  |
| Protease activity | |  |  |  |  |  |
| EW996827.1 | elastase 2* | 490 | AAI63342 | 6.15E-26 | 3.66 | 5.79 |
| CO870226.1 | transmembrane protease, serine 9 | 497 | AAI58103 | 1.78E-17 | 1.74 | 2.15 |
| EW997560.1 | zinc metalloprotease nas-14 | 334 | XP_002203097 | 1.41E-06 | 2.13 | 2.96 |
|  |  |  |  |  |  |  |
| protease inhibitor | |  |  |  |  |  |
| CK989145.1 | cystatin b | 624 | XP_002197519 | 1.51E-20 | 4.98 | 3.90 |
| CK989049.1 | kazal-type proteinase inhibitor | 502 | ACU46739 | 5.44E-09 | 2.40 | 2.77 |
|  |  |  |  |  |  |  |
| Misc |  |  |  |  |  |  |
| CO654068.1 | solute carrier family 22 (organic cation transporter) member 21 | 248 | XP_002738982 | 2.71E-12 | 5.22 | 2.85 |
| CV548593.1 | retrotransposon-like family member (retr-1)-like | 477 | XP_001203881 | 8.40E-22 | 1.98 | 1.89 |
| CV548679.1 | calcium alpha subunit family member (cca-1) | 617 | AAO83843 | 6.43E-69 | 1.88 | 2.18 |
| EW997078.1 | leucine-rich repeat contains calponin homology domain* | 588 | CAL52885 | 1.31E-04 | 3.48 | 3.67 |
| EW997416.1 | BclA | 649 | AAY15452 | 1.94E-13 | 2.41 | 2.94 |
| CK989050.1 | heterogeneous nuclear ribonucleoprotein a2 b1 homolog | 552 | CAX77145 | 9.25E-28 | 1.94 | 1.72 |
| CK988877.1 | myoglobin | 663 | O77003 | 4.80E-79 | 1.66 | 1.51 |
| EW997448.1 | adp/atp translocase 2 | 720 | XP_001455739 | 2.65E-52 | 1.81 | 2.72 |
| CK149190.1 | dihydropyrimidine dehydrogenase | 631 | CAM14145 | 4.92E-67 | 1.78 | 1.89 |
| CK149215.1 | dihydropyrimidine dehydrogenase | 666 | CAM14145 | 1.85E-70 | 1.94 | 2.05 |
| CV042555.1 | endo-beta-1,4 -glucanase | 220 | BAD44734 | 1.00E-15 | 11.11 | 5.61 |
| CK988663.1 | glucosaminyl (n-acetyl) transferase mucin type | 655 | EDL26188 | 5.05E-17 | 1.62 | 1.43 |
|  |  |  |  |  |  |  |
| Acc No. | Sequence desc. | bp | Hit ACC | E-Value | fc (RC/SC) | fc (RE/SE) |
| Resistant exposed only | |  |  |  |  |  |
| Immune/stress response | |  |  |  |  |  |
| CK989315.1 | FREP 3 | 587 | AAO59915 | 3.60E-57 | 1.29 | 1.33 |
| AF515464.1 | FREP12 | 2288 | AAO59918 | 0 | 1.42 | 1.95 |
| EW996829.1 | heat shock protein | 505 | XP_002231531 | 1.47E-09 | 1.37 | 1.82 |
| CK656707.1 | heat shock protein 70* | 513 | ACI90341 | 3.93E-77 | 1.66 | 2.83 |
| CK656737.1 | heat shock protein 70* | 516 | ACI90341 | 1.65E-68 | 1.82 | 3.12 |
| CK136129.1 | heat shock protein 70kda | 386 | AAB18178 | 5.01E-37 | 1.08 | 1.38 |
| CV548445.1 | heat shock protein 90 | 671 | ACC43967 | 1.06E-81 | 1.76 | 3.55 |
| EW997436.1 | nudix (nucleoside diphosphate linked moiety x)-type motif 15 | 609 | AAH64607 | 5.96E-11 | 1.02 | 1.54 |
| EW996984.1 | acyloxyacyl hydrolase | 580 | XP_574041 | 8.31E-41 | 1.06 | 1.63 |
|  |  |  |  |  |  |  |
| Antioxidant |  |  |  |  |  |  |
| CV548486.1 | peroxinectin | 768 | XP_002220096 | 3.00E-19 | 1.33 | 1.97 |
| EW997425.1 | peroxiredoxin 6 | 598 | XP_002109404 | 1.02E-28 | 1.22 | 1.78 |
| EW996707.1 | mitochondrial superoxide dismutase 2 variant 1 | 445 | AAS83980 | 2.26E-44 | 1.18 | 1.94 |
|  |  |  |  |  |  |  |
| Cytokeleton |  |  |  |  |  |  |
| EW996860.1 | tropomyosin | 400 | BAH10148 | 1.05E-33 | 1.23 | 2.59 |
| EW997342.1 | actin | 299 | BAD02941 | 8.83E-41 | 1.35 | 2.69 |
| EW997195.1 | alpha actin | 368 | ACN66629 | 8.70E-33 | 1.03 | 1.87 |
| EW997421.1 | alpha actin | 230 | ACN66629 | 1.74E-28 | -1.02 | 2.22 |
| EW996810.1 | alpha tubulin | 426 | CAX72946 | 1.28E-31 | 1.13 | 1.91 |
| EW997163.1 | beta 2 tubulin | 799 | NP_001032487 | 1.39E-111 | 1.12 | 1.92 |
| EW997522.1 | myosin essential light chain | 311 | ACC43966 | 6.69E-25 | -1.01 | 2.23 |
| CK149230.1 | myosin heavy chain | 706 | AAC46490 | 1.02E-42 | 1.29 | 1.41 |
| CV022404.1 | myosin heavy chain | 576 | CAB64662 | 2.40E-42 | 1.48 | 1.94 |
| CV548431.1 | myosin heavy chain | 726 | ACD68201 | 2.77E-73 | 1.02 | 2.58 |
| EW997386.1 | myosin heavy chain | 439 | XP_001607303 | 5.45E-35 | 1.16 | 3.02 |
| EW997462.1 | myosin heavy chain | 563 | 2EC6-A | 3.65E-75 | -1.01 | 2.43 |
| EW996818.1 | mitotic apparatus protein p62 | 729 | P91753 | 5.03E-06 | 1.16 | 1.46 |
|  |  |  |  |  |  |  |
| filaments |  |  |  |  |  |  |
| CK656726.1 | cytoplasmic intermediate filament protein* | 400 | CAA60122 | 7.06E-22 | 1.07 | 2.31 |
| CV022409.1 | intermediate filament protein | 665 | AAZ39528 | 4.79E-87 | 1.49 | 1.98 |
| EW996897.1 | Nestin | 699 | XP_001526331 | 3.03E-04 | 1.26 | 2.06 |
|  |  |  |  |  |  |  |
| Mitochondrial respiratory chain | |  |  |  |  |  |
| EW996850.1 | cytochrome b | 474 | AAQ74246 | 2.90E-63 | 1.73 | 3.06 |
| EW996859.1 | cytochrome b | 306 | YP_002808600 | 2.40E-06 | 1.11 | 1.46 |
| EW996954.1 | cytochrome b | 682 | AAQ74246 | 4.24E-102 | 1.70 | 3.63 |
| EW997260.1 | cytochrome b | 789 | YP_003692 | 5.44E-90 | 1.92 | 3.61 |
| EW997365.1 | cytochrome b | 273 | YP_002808600 | 1.08E-06 | 1.13 | 1.43 |
| EW997412.1 | cytochrome b* | 816 | AAK26394 | 4.59E-49 | 1.19 | 2.44 |
| EW997640.1 | cytochrome b | 765 | AAQ74246 | 4.59E-101 | 1.87 | 4.36 |
| EW997156.1 | cytochrome b5 | 384 | CAD92095 | 6.11E-18 | 1.03 | 1.50 |
| CK988958.1 | cytochrome c oxidase subunit i | 677 | NP_976176 | 2.14E-82 | 1.59 | 2.54 |
| EW996795.1 | cytochrome c oxidase subunit i | 726 | YP_654376 | 2.09E-78 | 1.22 | 2.23 |
| EW996858.1 | cytochrome c oxidase subunit i | 263 | NP_976176 | 1.17E-24 | 1.48 | 2.52 |
| EW996912.1 | cytochrome c oxidase subunit i | 578 | NP_976176 | 1.09E-85 | 1.57 | 2.60 |
| EW997025.1 | cytochrome c oxidase subunit i | 740 | NP_976176 | 2.50E-101 | 1.32 | 1.97 |
| EW997127.1 | cytochrome c oxidase subunit i* | 558 | CAX63228 | 2.46E-41 | 1.20 | 1.88 |
| EW997164.1 | cytochrome c oxidase subunit i | 754 | NP_976176 | 1.63E-103 | 1.30 | 1.87 |
| EW997198.1 | cytochrome c oxidase subunit i | 310 | NP_976176 | 1.50E-32 | 1.27 | 1.99 |
| EW997267.1 | cytochrome c oxidase subunit i | 338 | NP_976176 | 4.63E-38 | 1.44 | 2.39 |
| EW997305.1 | cytochrome c oxidase subunit i | 359 | NP_976176 | 9.06E-46 | 1.39 | 2.16 |
| EW997309.1 | cytochrome c oxidase subunit i | 287 | NP_976176 | 2.70E-29 | 1.48 | 2.78 |
| EW997388.1 | cytochrome c oxidase subunit i | 551 | NP_976176 | 2.44E-81 | 1.42 | 2.40 |
| EW997477.1 | cytochrome c oxidase subunit i | 773 | NP_976176 | 3.94E-108 | 1.31 | 1.88 |
| EW997517.1 | cytochrome c oxidase subunit i | 359 | NP_976176 | 2.17E-41 | 1.38 | 2.36 |
| CK989020.1 | cytochrome c oxidase subunit ii | 651 | NP_976169 | 6.89E-89 | 1.40 | 2.80 |
| CN445822.1 | cytochrome c oxidase subunit ii | 275 | NP_976169 | 4.99E-28 | 1.40 | 2.87 |
| EW996787.1 | cytochrome c oxidase subunit ii | 324 | NP_976169 | 1.56E-45 | 1.14 | 1.80 |
| EW996948.1 | cytochrome c oxidase subunit ii | 301 | NP_976169 | 2.57E-32 | 1.28 | 2.38 |
| EW997182.1 | cytochrome c oxidase subunit ii | 135 | YP_001648717 | 3.14E-06 | 1.26 | 2.61 |
| CK989929.1 | cytochrome c oxidase subunit iii | 401 | AAF31680 | 7.08E-13 | 1.17 | 1.83 |
| EW996939.1 | cytochrome c oxidase subunit iii | 500 | YP_002726387 | 1.62E-45 | 1.61 | 3.03 |
| EW997291.1 | cytochrome c oxidase subunit iii | 429 | AAQ74252 | 3.68E-47 | 1.58 | 2.95 |
| CO870242.1 | cytochrome oxidase subunit 1 | 670 | NP_976176 | 1.92E-99 | 1.50 | 2.46 |
| CV022390.1 | cytochrome oxidase subunit 1 | 334 | NP_976176 | 1.66E-15 | 1.43 | 2.20 |
| CK989495.1 | cytochrome oxidase subunit i | 245 | ABN50787 | 2.10E-20 | 1.35 | 2.01 |
| EW996782.1 | nadh dehydrogenase subunit 1 | 546 | NP_976166 | 1.08E-47 | 1.13 | 1.78 |
| EW996875.1 | nadh dehydrogenase subunit 1 | 691 | NP_976166 | 7.76E-83 | 1.56 | 2.25 |
| EW996895.1 | nadh dehydrogenase subunit 1 | 670 | NP_976166 | 6.00E-58 | 1.71 | 2.70 |
| EW997000.1 | nadh dehydrogenase subunit 1 | 771 | NP_976166 | 6.39E-77 | 1.73 | 3.39 |
| EW997265.1 | nadh dehydrogenase subunit 1 | 740 | NP_976166 | 8.61E-86 | 1.63 | 2.85 |
| EW997339.1 | nadh dehydrogenase subunit 1 | 756 | NP_976166 | 7.42E-88 | 1.71 | 3.30 |
| CK990026.1 | nadh dehydrogenase subunit 3 | 304 | NP_976172 | 2.25E-04 | 1.60 | 1.99 |
| CK988759.1 | nadh dehydrogenase subunit 4l | 341 | NP_976167 | 5.86E-13 | 1.32 | 1.69 |
| CV548266.1 | nadh dehydrogenase subunit 5 | 458 | NP_976165 | 1.26E-47 | 1.40 | 1.96 |
| EW997051.1 | mitochondrial cytochrome c oxidase subunit 5b isoform 1* | 537 | XP_001950983 | 3.29E-19 | 1.23 | 2.07 |
| EW997392.1 | atp synthase, H+ transporting, mitochondrial F0 subunit G | 471 | ACQ58142 | 7.25E-19 | 1.34 | 1.67 |
| CK988914.1 | atp synthase f0 subunit 6 | 513 | NP_976171 | 1.24E-30 | 1.32 | 2.32 |
| EW997569.1 | h+ transporting atp synthase o subunit* | 453 | NP_001040526 | 3.71E-15 | 1.21 | 2.33 |
| EW997559.1 | electron-transfer-flavoprotein beta polypeptide | 770 | XP_002412689 | 1.42E-41 | 1.10 | 1.59 |
|  |  |  |  |  |  |  |
| Signal transducer | |  |  |  |  |  |
| CO870195.1 | 14-3-3 protein | 363 | Q26537 | 1.95E-16 | 1.33 | 2.29 |
| EW996992.1 | 14-3-3 protein | 335 | Q26537 | 7.46E-16 | 1.35 | 2.39 |
| CK656730.1 | G protein coupled receptor kinase type 2 | 713 | XP_002422801 | 3.03E-42 | 1.21 | 2.06 |
| CV548512.1 | inhibitor of apoptosis protein 1 | 676 | XP_001957942 | 9.51E-06 | 1.41 | 1.97 |
| CK327232.1 | twitchin | 152 | BAC00784 | 1.58E-07 | 1.42 | 1.48 |
| EW997172.1 | elongation factor ef-1 alpha subunit | 523 | P28295 | 8.82E-64 | 1.32 | 2.18 |
| CK988691.1 | NFkB inhibitor | 474 | ABL74452 | 1.31E-19 | 1.40 | 1.48 |
| DW474843.1 | Atherin | 606 | NP_001071920 | 7.17E-17 | 1.11 | 1.87 |
| CK656742.1 | two pore segment channel 2 | 703 | AAI29068 | 7.42E-28 | 1.14 | 1.59 |
| CV548509.1 | titin | 570 | XP_001848911 | 4.55E-04 | 1.17 | 3.17 |
|  |  |  |  |  |  |  |
| Transcription regulator | |  |  |  |  |  |
| EW997166.1 | protein inhibitor of activated STAT 4 | 615 | XP_001374155 | 2.83E-24 | 1.13 | 1.63 |
| EW997174.1 | ESF1, nucleolar pre-rRNA processing protein | 591 | XP_002214687 | 6.15E-18 | 1.03 | 1.59 |
| CV548466.1 | g-box binding factor | 724 | AAA21021 | 4.62E-04 | 1.46 | 2.46 |
| CK656740.1 | bicoid-interacting protein 3 | 259 | XP_789930 | 9.00E-09 | 1.17 | 1.29 |
|  |  |  |  |  |  |  |
| Transcription |  |  |  |  |  |  |
| EW996989.1 | ski interacting protein | 570 | NP_001017145 | 5.11E-56 | -1.02 | 1.59 |
| EW996727.1 | elongation protein 4 homolog | 437 | XP_001180734 | 1.35E-25 | 1.17 | 1.92 |
|  |  |  |  |  |  |  |
| Translation regulation | |  |  |  |  |  |
| CV548482.1 | cug triplet rna binding protein 1 | 632 | CAG10818 | 3.89E-12 | 1.64 | 2.47 |
|  |  |  |  |  |  |  |
| Translation/protein synthesis | |  |  |  |  |  |
| EW996806.1 | ribosomal protein l10 | 509 | ABM55536 | 6.64E-66 | 1.40 | 2.35 |
| EW997549.1 | ribosomal protein l35 | 331 | Q90YT4 | 3.14E-14 | 1.22 | 1.86 |
| EW996797.1 | ribosomal protein l7a | 889 | NP_001153326 | 2.02E-64 | 1.29 | 1.94 |
| EW997065.1 | ribosomal protein s12 | 383 | AAY66899 | 1.61E-34 | 1.21 | 1.56 |
| EW997186.1 | ribosomal protein s27a | 319 | NP_990284 | 1.53E-16 | 1.23 | 1.59 |
| CO870194.1 | 40s ribosomal protein s4 | 458 | ABR23501 | 4.47E-45 | 1.05 | 1.89 |
| DW474520.1 | mitochondrial ribosomal protein s2 | 381 | XP_002581095 | 1.16E-08 | 1.13 | 1.64 |
| CV548437.1 | elongation factor 2 | 555 | AAQ77163 | 1.22E-59 | 1.20 | 2.01 |
| EW997067.1 | elongation factor 2* | 382 | ACN58670 | 5.39E-06 | 1.30 | 3.00 |
| EW997555.1 | elongation factor 2* | 491 | NP_001015785 | 3.48E-29 | 1.45 | 2.69 |
| EW996824.1 | adenosylhomocysteinase 2* | 834 | XP_002129385 | 1.61E-30 | 1.08 | 1.78 |
| CK656696.1 | histidyl-tRNA synthetase* | 725 | XP_002191168 | 1.12E-66 | 1.57 | 2.07 |
| CK656731.1 | histidyl-tRNA synthetase | 674 | NP_001020585 | 1.97E-51 | 1.52 | 1.94 |
| EW997131.1 | tyrosyl-tRNA synthetase* | 507 | XP_397348 | 1.54E-30 | 1.13 | 2.02 |
| CO870190.1 | glutamyl-prolyl tRNA synthetase* | 694 | XP_001994240 | 4.50E-38 | 1.52 | 2.43 |
|  |  |  |  |  |  |  |
| Protein folding and translocation | |  |  |  |  |  |
| EW997568.1 | Dynein, light roadblock-type 2 | 468 | NP_001155557 | 1.21E-05 | 1.26 | 1.62 |
| EW997461.1 | Clathrin | 865 | EDM05574 | 4.33E-125 | 1.04 | 1.85 |
| EW997020.1 | importin alpha-like protein | 198 | XP_001958292 | 2.68E-13 | 1.35 | 1.58 |
| EW997022.1 | vesicle-associated membrane protein 3 | 307 | XP_001375285 | 5.15E-17 | 1.30 | 2.09 |
| CK656703.1 | Sequestosome-1* | 640 | XP_002428257 | 2.23E-05 | 1.11 | 2.06 |
| CK656744.1 | exportin crm1 homolog | 675 | XP_001604619 | 2.31E-93 | 1.37 | 2.22 |
| EW996761.1 | flotillin 2 | 355 | XP_002215587 | 6.73E-49 | 1.08 | 1.72 |
| EW997419.1 | myotubularin-related protein 2 | 320 | XP_002131095 | 1.09E-30 | 1.05 | 1.37 |
| EW997074.1 | protein disulfide isomerase | 687 | NP_998529 | 1.48E-09 | 1.08 | 1.41 |
|  |  |  |  |  |  |  |
| Protein degredation | |  |  |  |  |  |
| CK656741.1 | ubiquitin* | 369 | XP_001602185 | 3.09E-25 | 1.31 | 2.72 |
| CK656733.1 | ubiquitin-conjugating enzyme e2d 2 (ubc4 5 yeast)* | 683 | ACO13579 | 1.11E-17 | 1.35 | 2.34 |
| DW474102.1 | homocysteine- endoplasmic reticulum stress- ubiquitin-like domain member 1 | 412 | NP_998846 | 1.72E-04 | -1.09 | 1.42 |
| EW997041.1 | Proteasome 26s atpase complex subunit 4 | 276 | XP_002200588 | 9.05E-30 | 1.14 | 1.81 |
|  |  |  |  |  |  |  |
| Protease activity | |  |  |  |  |  |
| EW996801.1 | cathepsin l | 591 | XP_002161512 | 1.77E-49 | 1.26 | 2.75 |
| EW997422.1 | peptidase c1a papain | 545 | CAM77442 | 3.02E-12 | 1.13 | 1.28 |
| CO870213.1 | transmembrane protease, serine 9 | 497 | AAI58103 | 3.96E-17 | 1.53 | 1.73 |
|  |  |  |  |  |  |  |
| Glycolysis |  |  |  |  |  |  |
| DW474741.1 | phosphofructokinase | 518 | XP_966779 | 5.55E-15 | 1.05 | 1.77 |
| EW996863.1 | pyruvate dehydrogenase protein x mitochondrial precursor | 423 | XP_001945646 | 7.80E-24 | 1.16 | 1.39 |
|  |  |  |  |  |  |  |
| Citric acid cycle | |  |  |  |  |  |
| EW997411.1 | malate dehydrogenase* | 320 | XP_641333 | 4.04E-09 | 1.02 | 1.99 |
|  |  |  |  |  |  |  |
| Blood constituents | |  |  |  |  |  |
| CN476075.1 | haemoglobin type 1 | 750 | CAJ44466 | 1.54E-97 | 1.71 | 2.35 |
| CV174015.1 | haemoglobin type 1 | 640 | CAJ44466 | 7.50E-63 | 1.83 | 2.44 |
| CV548291.1 | haemoglobin type 1 | 854 | CAJ44466 | 4.48E-151 | 1.75 | 2.53 |
| CK989867.1 | Coagulation factor XI (serine protease) | 558 | XP_002215683 | 3.91E-13 | 1.32 | 1.51 |
|  |  |  |  |  |  |  |
| Nucleoproteins | |  |  |  |  |  |
| EW997529.1 | obg GTPase type protein | 213 | XP_002573039 | 9.07E-14 | 1.37 | 1.99 |
| EW997527.1 | histone h3 | 293 | XP_001928622 | 2.97E-28 | 1.60 | 2.04 |
|  |  |  |  |  |  |  |
| Misc |  |  |  |  |  |  |
| EW996702.1 | Ag [Aplysia californica] | 451 | AAB17098 | 5.52E-05 | 1.08 | 2.00 |
| EW997557.1 | ankyrin unc44 | 515 | AAO25692 | 1.38E-13 | 1.28 | 1.71 |
| CV548449.1 | tetratricopeptide repeat domain | 318 | ACC43969 | 2.61E-16 | 1.27 | 2.18 |
| CK656706.1 | viral a-type inclusion protein* | 673 | XP_001582797 | 1.25E-05 | 1.13 | 1.57 |
| CK656612.1 | zinc finger protein | 473 | XP_785557 | 6.57E-12 | 1.09 | 1.82 |
| CV548603.1 | endonuclease reverse transcriptase | 308 | XP_001196407 | 4.95E-06 | 1.39 | 1.47 |
| DY523268.1 | lin-24 (twenty-four) like family member (lntl-1) | 667 | XP_002578591 | 6.67E-20 | 1.15 | 2.63 |
| EW996744.1 | fimbriae-associated protein | 744 | XP_001200765 | 1.53E-04 | 1.19 | 1.63 |
| EW996808.1 | nhl repeat containing protein* | 483 | ACD54679 | 8.93E-33 | 1.25 | 1.94 |
| DW474244.1 | flavin reductase | 638 | XP_002240105 | 1.41E-21 | 1.04 | 1.62 |
| EW997519.1 | carbonic anhydrase XV c | 449 | NP_001070086 | 3.59E-12 | 1.27 | 1.74 |
| CV548425.1 | o-acyltransferase (membrane bound) domain containing 2 | 325 | XP_002197683 | 1.74E-04 | 1.12 | 1.38 |
| DW474096.1 | cellulase | 317 | BAH85844 | 1.94E-11 | 1.44 | 1.68 |
| EW997572.1 | pancreatic triacylglycerol lipase | 715 | XP_002238459 | 9.46E-18 | 1.28 | 2.62 |
|  |  |  |  |  |  |  |
| Susceptible control only | |  |  |  |  |  |
| Acc No | Sequence desc. | bp | Hit ACC | E-Value | fc (RC/SC) | fc (RE/SE) |
| Stress response/ innate defence | |  |  |  |  |  |
| CK989857.1 | antimicrobial peptide hydramacin | 549 | A5GZY1 | 3.48E-11 | -1.52 | -1.12 |
| EW997079.1 | heat shock protein 60 | 156 | ACL00842 | 3.98E-19 | -1.56 | -1.13 |
|  |  |  |  |  |  |  |
| Antioxidant |  |  |  |  |  |  |
| DW474756.1 | Thioredoxin peroxidase 3 | 533 | XP_002571905 | 2.33E-38 | -1.58 | -1.06 |
| EW996752.1 | thioredoxin peroxidase 2 | 513 | ABO26635 | 9.41E-55 | -1.32 | -1.00 |
|  |  |  |  |  |  |  |
| Cytoskeletal |  |  |  |  |  |  |
| CK656684.1 | Tubulin beta 2c | 314 | BAF46873 | 5.45E-38 | -1.95 | -1.60 |
| CK656671.1 | tubulin beta-1 chain | 692 | NP_001036964 | 9.19E-76 | -1.31 | -1.11 |
| CV548104.1 | Fermitin | 677 | XP_002168557 | 2.75E-29 | -1.92 | -1.33 |
| CK640673.1 | mitofusin 2 | 534 | XP_002218605 | 1.05E-26 | -1.47 | -1.24 |
|  |  |  |  |  |  |  |
| Mitochondrial respiratory chain | |  |  |  |  |  |
| EW996922.1 | cytochrome c | 636 | P00040 | 1.77E-48 | -1.89 | -1.45 |
| EW997647.1 | NADH ubiqinone dehydrogenase | 672 | XP_973540 | 1.79E-28 | -1.39 | -1.20 |
| CK989465.1 | mitochondrial complex i subunit ndufa7 | 577 | XP_975169 | 1.78E-11 | -1.65 | -1.35 |
|  |  |  |  |  |  |  |
| Signal transduction | |  |  |  |  |  |
| CO870367.1 | rho gtpase activating protein 28 | 377 | EDL38339 | 1.51E-10 | -2.48 | -1.88 |
| CV548649.1 | rho gtpase-activating protein 28 isoform 2 | 357 | XP_002757111 | 9.70E-10 | -2.40 | -1.90 |
| EG030742.1 | IKAP | 666 | XP_002110439 | 6.64E-36 | -1.40 | -1.31 |
| CO635887.1 | Testis-specific serine kinase 1 | 430 | XP_002119260 | 1.51E-24 | -2.29 | -1.51 |
| CK989213.1 | phosphoglycerate kinase 1 | 522 | O61471 | 1.85E-37 | -1.59 | -1.36 |
| CV548335.1 | phosphoglycerate kinase 1 | 730 | O61471 | 1.72E-22 | -1.97 | -1.71 |
| CV548397.1 | phosphoglycerate kinase 1 | 318 | O61471 | 7.10E-22 | -1.86 | -1.57 |
| CV042533.1 | GTP binding nuclear protein RAN | 422 | ACV66339 | 2.86E-39 | -1.70 | -1.35 |
|  |  |  |  |  |  |  |
| Transcription processes | |  |  |  |  |  |
| CV548108.1 | cleavage and polyadenylation specific factor 2 | 462 | XP_394940 | 3.86E-52 | -1.42 | -1.22 |
| DW473992.1 | cyclin a | 569 | P04962 | 3.32E-34 | -1.73 | -1.43 |
| EW996734.1 | endonuclease-reverse transcriptase | 302 | XP_002570337 | 1.12E-19 | -1.51 | -1.22 |
|  |  |  |  |  |  |  |
| Transcription regulator | |  |  |  |  |  |
| DW474600.1 | thyroid hormone receptor interactor 11 | 577 | XP_537351 | 4.80E-33 | -1.66 | -1.30 |
|  |  |  |  |  |  |  |
| Translation processes | |  |  |  |  |  |
| CK989120.1 | eukaryotic translation initiation factor subunit k | 554 | XP_002242431 | 1.69E-29 | -1.54 | -1.23 |
| EW997415.1 | eukaryotic translation initiation factor subunit k | 561 | NP_001017583 | 1.35E-45 | -1.38 | -1.32 |
| CV548339.1 | exosome component 9 | 751 | ACH87550 | 2.87E-84 | -1.91 | -1.48 |
|  |  |  |  |  |  |  |
| Protein folding and translocation | |  |  |  |  |  |
| CO870284.1 | fk506 binding protein 59kda | 586 | XP_002191051 | 4.42E-21 | -2.31 | -1.46 |
| CV548730.1 | fk506 binding protein 59kda | 586 | XP_002191051 | 4.42E-21 | -2.20 | -1.58 |
|  |  |  |  |  |  |  |
| Protein degredation | |  |  |  |  |  |
| DW474627.1 | Ubiquitin conjugating enzyme E2 | 584 | XP_001350824 | 1.90E-08 | -2.03 | -1.25 |
| CV042593.1 | Ubiquitin-conjugating enzyme e2d 3 | 329 | XP_001368675 | 4.82E-55 | -1.61 | -1.37 |
| CK989172.1 | sumo1 activating enzyme subunit 1 | 529 | XP_002241538 | 6.11E-23 | -1.57 | -1.35 |
|  |  |  |  |  |  |  |
| Serine peptidase inhibitor | |  |  |  |  |  |
| CK988862.1 | secreted protein with signal peptide and 12 kazal repeats and a mucin-like stretch of threonines | 434 | XP_625931 | 1.41E-06 | -1.52 | -1.12 |
|  |  |  |  |  |  |  |
| Protease activity | |  |  |  |  |  |
| CO870281.1 | methionine aminopeptidase | 367 | XP_002432191 | 2.54E-24 | -1.50 | -1.20 |
|  |  |  |  |  |  |  |
| Replication |  |  |  |  |  |  |
| CO870252.1 | mutl homolog | 396 | EDL77036 | 1.53E-24 | -1.50 | -1.24 |
|  |  |  |  |  |  |  |
| Misc |  |  |  |  |  |  |
| CV548657.1 | glycerol-3-phosphate dehydrogenase | 700 | AAC32663 | 8.56E-77 | -1.64 | -1.19 |
| CK989459.1 | perilipin 2 | 496 | NP_988963 | 1.18E-07 | -1.65 | -1.51 |
| CO870344.1 | iq motif containing with aaa domain | 488 | XP_780770 | 1.06E-17 | -1.71 | -1.27 |
| CV548165.1 | phospholipase c beta | 522 | XP_002227771 | 3.64E-09 | -1.65 | -1.34 |
| DW474691.1 | triosephosphate isomerase glyceraldehyde-3-phosphate dehydrogenase | 530 | ABU99333 | 3.07E-51 | -2.12 | -1.53 |
|  |  |  |  |  |  |  |
| Susceptible control and exposed | |  |  |  |  |  |
| Acc No | Sequence desc. | bp | Hit ACC | E-Value | fc (RC/SC) | fc (RE/SE) |
| Stress response/innate defence | |  |  |  |  |  |
| CK989131.1 | antimicrobial peptide hydramacin | 541 | A5GZY1 | 4.43E-16 | -2.02 | -1.63 |
| CK989806.1 | antimicrobial peptide hydramacin | 512 | 2K35-A | 3.21E-07 | -1.82 | -1.58 |
| CO654048.1 | Nop14 | 450 | XP_536223 | 7.43E-18 | -1.60 | -1.48 |
| EE723682.1 | hydroxyacyl-coenzyme a dehydrogenase 3-ketoacyl-coenzyme a thiolase enoyl-coenzyme a hydratase (trifunctional protein) alpha subunit | 169 | XP_001605350 | 1.98E-08 | -1.43 | -1.46 |
| CK989482.1 | neuromacin | 425 | A5GZY1 | 1.29E-11 | -2.11 | -1.78 |
|  |  |  |  |  |  |  |
| Antioxidants |  |  |  |  |  |  |
| DW473897.1 | thioredoxin domain containing 12 (endoplasmic reticulum) | 667 | XP_001635954 | 2.70E-29 | -2.15 | -2.22 |
| DW473928.1 | thioredoxin domain containing 12 (endoplasmic reticulum) | 668 | NP_001139851 | 9.93E-32 | -2.57 | -2.86 |
|  |  |  |  |  |  |  |
| Cytoskeletal |  |  |  |  |  |  |
| CO870278.1 | fructose 1, 6 biphosphate adolase | 777 | ABY87348 | 3.28E-62 | -3.12 | -3.02 |
| CO870355.1 | fructose 1, 6 biphosphate adolase | 782 | ABY87348 | 2.80E-61 | -2.74 | -3.06 |
| CK656607.1 | beta-tubulin | 567 | NP_001032345 | 1.55E-73 | -2.20 | -2.14 |
| CK149463.1 | EF-hand containing protein | 496 | CAX73306 | 2.84E-07 | -1.65 | -1.79 |
|  |  |  |  |  |  |  |
| Signal transduction | |  |  |  |  |  |
| CO870310.1 | testis-specific serine kinase 6 | 551 | XP_002217679 | 4.52E-19 | -3.06 | -2.49 |
| CV548765.1 | phosphoglycerate kinase 1 | 318 | O61471 | 7.09E-22 | -1.89 | -1.60 |
| CK149239.1 | inhibitor of apoptosis protein | 657 | CAG00512 | 1.49E-16 | -1.90 | -1.85 |
| CK149394.1 | Unknown protein contains ITAM | 580 | XP_001183214 | 4.28E-21 | -2.17 | -2.06 |
| CK989782.1 | synaptojanin 2 binding protein | 402 | BAA76285 | 1.38E-09 | -1.70 | -1.72 |
| CO870313.1 | rho gtpase activating protein 28 | 362 | XP_512034 | 5.72E-08 | -2.33 | -2.13 |
| CO870322.1 | rho gtpase-activating protein 28 isoform 2 | 328 | XP_002757111 | 7.45E-10 | -2.49 | -2.27 |
| CO870419.1 | Fibrillin | 787 | XP_001632251 | 8.96E-15 | -1.62 | -2.46 |
|  |  |  |  |  |  |  |
| Transcriptional processes | |  |  |  |  |  |
| CN549171.1 | polymerase ii (dna directed) polypeptide a isoform 7 | 242 | XP_858007 | 3.57E-04 | -2.29 | -2.44 |
| CN779713.1 | polymerase ii (dna directed) polypeptide a isoform 7 | 252 | XP_858007 | 3.65E-04 | -2.23 | -2.31 |
| CK149584.1 | endonuclease-reverse transcriptase | 893 | AAL57609 | 9.23E-17 | -1.87 | -1.86 |
| CK989480.1 | GM26494 | 443 | XP_002032323 | 1.63E-04 | -1.62 | -1.97 |
| CK990123.1 | mki67 (fha domain) interacting nucleolar phosphoprotein | 549 | XP_422088 | 1.32E-18 | -1.51 | -1.64 |
| CV548771.1 | nuclear receptor subfamily group member 2 | 715 | ABU89803 | 5.87E-52 | -1.50 | -1.63 |
| CK149435.1 | methenyltetrahydrofolate synthetase domain containing | 682 | XP_002214657 | 2.88E-58 | -1.60 | -1.50 |
| EW996836.1 | polyprotein | 610 | BAA35104 | 2.43E-04 | -17.71 | -17.39 |
| EW996820.1 | polyprotein | 800 | ACI47517 | 9.67E-11 | -26.26 | -23.79 |
| EW997437.1 | polyprotein | 512 | BAA35104 | 1.75E-04 | -24.17 | -25.02 |
|  |  |  |  |  |  |  |
| Translation regulation | |  |  |  |  |  |
| CK149373.1 | staufen | 257 | AAO38741 | 2.53E-27 | -1.75 | -1.72 |
| EW997479.1 | neuron-specific staufen | 657 | AAW84263 | 5.30E-06 | -1.70 | -1.71 |
| CK149551.1 | eukaryotic translation initiation factor subunit 9 116kda | 751 | XP_002414993 | 1.33E-37 | -1.81 | -1.74 |
| EW997653.1 | eukaryotic translation initiation factor subunit c | 708 | CAG07597 | 8.67E-24 | -1.76 | -1.63 |
|  |  |  |  |  |  |  |
| translation |  |  |  |  |  |  |
| EE723175.1 | ribosomal protein l2 | 111 | XP_002435962 | 3.21E-06 | -2.35 | -1.72 |
| CK988978.1 | ribosomal protein l35a | 675 | ABZ04243 | 1.60E-53 | -1.54 | -1.79 |
| CV548375.1 | ribosomal protein s6 | 633 | Q9BMX5 | 3.32E-23 | -1.65 | -1.58 |
| EW997649.1 | mitochondrial ribosomal protein S24 | 497 | ABO26683 | 2.40E-22 | -1.60 | -1.54 |
| CK988782.1 | mitochondrial ribosomal protein l42 | 480 | XP_001139409 | 1.89E-11 | -1.91 | -1.60 |
| CK989201.1 | nucleolar protein family member 2 | 602 | XP_625159 | 5.35E-09 | -1.55 | -1.55 |
|  |  |  |  |  |  |  |
| Protein folding and translocation | |  |  |  |  |  |
| CK989178.1 | grpE protein | 530 | XP_002425913 | 1.49E-21 | -1.96 | -1.69 |
| CV548360.1 | atp-binding sub-family f member 2 | 391 | AAH66505 | 7.88E-34 | -1.87 | -2.11 |
| CV548418.1 | atp-binding sub-family f member 2 | 468 | AAH66505 | 1.73E-36 | -1.79 | -2.03 |
| DW473989.1 | Dynein light tctex-type 1 | 501 | XP_783725 | 1.21E-16 | -1.53 | -1.58 |
| DW474490.1 | Dynein | 690 | XP_002572189 | 1.74E-20 | -1.93 | -2.18 |
| EE723159.1 | mitochondrial inner membrane protein translocase | 136 | XP_001658891 | 1.82E-08 | -1.82 | -1.64 |
| CV022380.1 | Pho-PQ, activated pathogenicity-related protein | 470 | XP_002213192 | 8.32E-23 | -1.97 | -1.99 |
| DW474452.1 | chaperonin containing TCP1, subunit 4 | 638 | XP_002209398 | 4.25E-31 | -1.94 | -1.54 |
| DW474387.1 | protein disulfide isomerase family member 4 | 521 | XP_791396 | 4.51E-04 | -2.51 | -2.69 |
| CV548373.1 | protein disulfide isomerase family member 4 | 623 | XP_001368451 | 5.83E-41 | -2.28 | -1.96 |
| DW474123.1 | vacuolar protein sorting 28 homolog | 504 | XP_002402690 | 2.04E-19 | -1.58 | -1.57 |
|  |  |  |  |  |  |  |
| Protein degredation | |  |  |  |  |  |
| CO870301.1 | ubiquitin c | 480 | CAX79713 | 8.32E-76 | -1.56 | -1.78 |
| CV548652.1 | ubiquitin-conjugating enzyme e2 | 737 | ACD13594 | 1.32E-70 | -1.55 | -1.48 |
| EW996970.1 | ubiquitin-conjugating enzyme e2i | 240 | XP_001640489 | 1.54E-15 | -1.52 | -1.44 |
| DW474587.1 | polyubiquitin | 588 | AAR32784 | 8.87E-65 | -1.68 | -1.87 |
| DW473949.1 | proteasome (macropain) beta 8 (large multifunctional protease 7) | 566 | XP_002211111 | 4.18E-18 | -2.14 | -1.94 |
| CV548287.1 | proteasome beta 5 subunit | 235 | XP_002211111 | 3.99E-17 | -1.84 | -1.79 |
| CK990004.1 | proteasome maturation protein | 612 | NP_001005093 | 8.17E-08 | -1.30 | -1.30 |
|  |  |  |  |  |  |  |
| Post translational modification? | |  |  |  |  |  |
| CK327235.1 | Su(var) 2 gene | 252 | XP_002434665 | 1.55E-08 | -2.28 | -1.91 |
|  |  |  |  |  |  |  |
| Cell proliferation | |  |  |  |  |  |
| CK149395.1 | endonuclease g | 664 | XP_415487 | 1.51E-32 | -2.22 | -3.20 |
| CK640679.1 | endonuclease g | 409 | XP_415487 | 1.01E-20 | -2.38 | -2.91 |
| CK149378.1 | endonuclease g | 364 | XP_002736286 | 1.59E-04 | -2.01 | -2.14 |
| CO870350.1 | endonuclease mitochondrial precursor | 662 | XP_002004840 | 3.82E-28 | -2.61 | -3.63 |
|  |  |  |  |  |  |  |
| Catalytic activity | |  |  |  |  |  |
| CF216478.1 | diacylglycerol lipase beta | 405 | XP_002197376 | 3.05E-17 | -1.44 | -1.41 |
| DW474845.1 | hras-like suppressor 3 | 642 | XP_002207573 | 5.68E-26 | -2.37 | -1.91 |
| CK989339.1 | Deoxyribonucleoside kinase domain containing | 525 | EFA00478 | 9.45E-04 | -1.51 | -1.44 |
|  |  |  |  |  |  |  |
| Misc |  |  |  |  |  |  |
| EW996739.1 | atp synthase-like protein | 773 | XP_002412695 | 3.28E-08 | -4.72 | -3.93 |
| CK989051.1 | c19orf66 protein | 574 | NP_001033270 | 5.26E-08 | -2.28 | -2.33 |
| CO870360.1 | glycerol-3-phosphate dehydrogenase | 633 | ABN12045 | 6.68E-32 | -1.68 | -1.61 |
| CK989939.1 | sam dependent methyltransferase | 569 | EFA74693 | 8.01E-04 | -1.78 | -2.23 |
| CK656846.1 | ChaC, cation transport regulator homolog 2 | 555 | NP_001026203 | 7.18E-44 | -1.56 | -1.60 |
| CV548610.1 | ChaC, cation transport regulator homolog 3 | 469 | NP_001026203 | 3.25E-43 | -1.45 | -1.53 |
| CK989129.1 | transmembrane protein 93 | 545 | XP_001508433 | 2.30E-28 | -1.68 | -1.70 |
| CV548241.1 | coiled-coil domain containing protein 65 | 695 | XP_001504180 | 1.44E-63 | -1.50 | -1.47 |
| CK149530.1 | rio kinase 3 | 669 | NP_001087045 | 6.92E-49 | -2.26 | -2.45 |
|  |  |  |  |  |  |  |
| Susceptible exposed only | |  |  |  |  |  |
| Acc No. | Sequence desc. | bp | Hit ACC | E-Value | fc (RC/SC) | fc (RE/SE) |
| Stress response | |  |  |  |  |  |
| CV548644.1 | coproporphyrinogen oxidase | 729 | AAP34327 | 1.44E-61 | -1.46 | -1.87 |
|  |  |  |  |  |  |  |
| protease inhibitor | |  |  |  |  |  |
| DW474235.1 | type 2 cystatin | 606 | AAV91521 | 2.09E-08 | -1.40 | -3.71 |
| DW474250.1 | type 2 cystatin | 595 | AAV91521 | 4.49E-08 | 1.71 | -2.77 |
|  |  |  |  |  |  |  |
| Antioxidants |  |  |  |  |  |  |
| CK149203.1 | dual oxidase 1 | 566 | XP_002205183 | 8.34E-51 | -1.42 | -2.13 |
| CK149399.1 | dyp-type peroxidase family protein | 640 | CAX74015 | 4.29E-34 | -1.72 | -1.88 |
| CV548307.1 | iron-dependent peroxidase | 767 | XP_002108063 | 3.84E-23 | -1.99 | -2.17 |
|  |  |  |  |  |  |  |
| Extracellular matrix | |  |  |  |  |  |
| CV548074.1 | fibropellin ia | 237 | XP_001178560 | 1.51E-16 | -1.10 | -3.37 |
|  |  |  |  |  |  |  |
| cytoskeleton |  |  |  |  |  |  |
| CV548251.1 | alpha tubulin | 301 | XP_001630129 | 4.09E-38 | -1.75 | -2.18 |
| EW996994.1 | alpha tubulin a1 | 232 | XP_002122739 | 5.55E-13 | -1.77 | -1.80 |
| CN476107.1 | alpha tubulin | 775 | CAX72946 | 7.35E-124 | -1.65 | -2.07 |
| CV548153.1 | myosin 3A | 702 | XP_002226652 | 2.09E-22 | -1.32 | -2.02 |
|  |  |  |  |  |  |  |
| Adhesion proteins | |  |  |  |  |  |
| CN445765.1 | egf-like domain-containing protein | 596 | AAZ80798 | 1.52E-64 | -1.24 | -2.04 |
| CO635925.1 | egf-like domain-containing protein | 420 | AAZ80798 | 3.18E-30 | -1.37 | -2.82 |
| CK149291.1 | agrin | 660 | EDL81364 | 5.96E-04 | -1.27 | -1.55 |
| CV548246.1 | agrin | 644 | EDL81364 | 4.68E-04 | -1.33 | -1.54 |
| CV548571.1 | agrin | 693 | EDL81364 | 5.56E-04 | -1.27 | -1.56 |
| CN476116.1 | tandem repeat galectin | 492 | ABS28869 | 1.26E-71 | -1.75 | -2.17 |
|  |  |  |  |  |  |  |
| Mitochondrial respiratory chain | |  |  |  |  |  |
| EW996985.1 | atp synthase h+ mitochondrial f0 subunit c3 (subunit 9) | 430 | ABY87376 | 1.46E-19 | -1.21 | -1.57 |
|  |  |  |  |  |  |  |
| Signal transduction | |  |  |  |  |  |
| CO870262.1 | dual specificity protein kinase | 682 | XP_001951299 | 1.50E-06 | -1.44 | -1.81 |
| CK800779.1 | ahnak nucleoprotein isoform 1 | 349 | AAQ97238 | 3.19E-14 | -1.55 | -2.40 |
| CK149387.1 | neuroblast differentiation-associated protein ahnak | 696 | XP_001623342 | 7.83E-06 | -1.39 | -2.17 |
| CK149540.1 | connective tissue growth factor | 542 | NP_001001826 | 4.56E-29 | -1.00 | -1.57 |
| CK989034.1 | cytidine deaminase | 667 | AAZ39529 | 1.12E-75 | -1.41 | -1.69 |
| DW474830.1 | guanine nucleotide exchange | 543 | XP_002142680 | 1.72E-07 | 1.24 | -2.48 |
| CV548677.1 | transforming growth beta receptor 1 | 741 | AAI70320 | 2.23E-57 | -1.05 | -1.35 |
|  |  |  |  |  |  |  |
| Transcription regulator | |  |  |  |  |  |
| CK656647.1 | female sterile homeotic isoform A | 655 | XP_002057394 | 5.39E-27 | -1.35 | -1.56 |
| CN476090.1 | matriptase | 307 | XP_001101446 | 3.35E-08 | 1.31 | -2.43 |
| CN779728.1 | zinc finger protein | 646 | XP_001193133 | 5.41E-08 | -1.10 | -1.51 |
| DW474560.1 | zinc finger protein | 600 | Q64213 | 1.32E-55 | -1.29 | -1.53 |
| EW996754.1 | zinc finger protein | 432 | XP_001201671 | 7.40E-10 | 1.02 | -1.75 |
|  |  |  |  |  |  |  |
| Transcriptional processes | |  |  |  |  |  |
| DW474166.1 | 5-aminoimidazole-4-carboxamide ribonucleotide formyltransferase imp cyclohydrolase | 576 | NP_001076265 | 3.32E-42 | -1.28 | -1.33 |
| DW474805.1 | heterogeneous nuclear ribonucleoprotein r | 570 | ABP04054 | 4.24E-10 | -1.12 | -1.48 |
| CK989306.1 | mec-8 | 527 | XP_001605039 | 7.99E-20 | -1.61 | -2.11 |
|  |  |  |  |  |  |  |
| RNA processing | |  |  |  |  |  |
| DW474015.1 | exosome component 7 | 592 | XP_002217737 | 3.30E-49 | -1.05 | -1.30 |
| CK988794.1 | small nuclear ribonucleoprotein polypeptide g | 551 | ACG76261 | 1.24E-22 | -1.59 | -1.78 |
| CO870273.1 | polyadenylate-binding protein | 399 | XP_396057 | 7.31E-19 | -1.01 | -1.59 |
|  |  |  |  |  |  |  |
| Translational regulator | |  |  |  |  |  |
| CK085023.1 | aconitase 1 | 245 | XP_002429734 | 7.43E-24 | -1.45 | -1.53 |
|  |  |  |  |  |  |  |
| Translational processes | |  |  |  |  |  |
| DW474286.1 | ribosomal protein l15 | 606 | XP_001624083 | 7.31E-62 | -1.63 | -1.69 |
| EW997040.1 | ribosomal protein s10 | 251 | CAD91124 | 6.25E-23 | -1.48 | -1.59 |
| CK988665.1 | ribosomal protein s20 | 663 | CAD91428 | 2.59E-16 | -1.28 | -1.42 |
| CN779691.1 | ribosomal protein s6 | 480 | Q9BMX5 | 1.35E-25 | -1.53 | -1.90 |
| EW996900.1 | ribosomal protein s6 | 688 | Q9BMX5 | 1.14E-94 | -1.31 | -1.49 |
| CK989949.1 | 40s ribosomal protein s10 | 488 | CAD91124 | 1.37E-41 | -1.32 | -1.66 |
| CK989079.1 | eukaryotic translation elongation factor 1 alpha 2 | 609 | ABD67497 | 9.98E-75 | -1.20 | -1.43 |
| AF179902.1 | eukaryotic translation initiation factor 6 | 341 | AAF07057 | 1.26E-15 | -1.43 | -1.46 |
|  |  |  |  |  |  |  |
| Protein folding and trafficking | |  |  |  |  |  |
| CK989816.1 | Endophilin | 609 | ABY87380 | 3.48E-19 | -1.23 | -1.50 |
| EW997472.1 | calreticulin | 458 | AAB24569 | 8.99E-62 | -1.61 | -1.91 |
| EW997576.1 | dynein heavy chain at 16f cg7092-pa | 355 | XP_001744190 | 5.78E-08 | -1.28 | -1.57 |
| CK149563.1 | translocon-associated protein subunit alpha | 419 | XP_002213048 | 5.09E-28 | -1.62 | -1.75 |
|  |  |  |  |  |  |  |
| Protein degredation | |  |  |  |  |  |
| CK149524.1 | ubiquitin c | 273 | CAX79713 | 1.22E-42 | -1.65 | -2.23 |
| CK989387.1 | ubiquitin c | 520 | NP_776558 | 1.39E-29 | -1.68 | -2.08 |
| EW997658.1 | proteasome (macropain) alpha 5 | 706 | XP_002217975 | 4.93E-49 | -1.80 | -1.90 |
| CK989932.1 | proteasome subunit alpha type-2 | 656 | ABO26645 | 5.14E-70 | -1.46 | -1.70 |
| CN779698.1 | proteasome subunit alpha type-2 | 469 | ABO26645 | 1.78E-49 | -1.76 | -2.29 |
|  |  |  |  |  |  |  |
| Cell proliferation | |  |  |  |  |  |
| CK656615.1 | endonuclease g | 295 | XP_851160 | 2.97E-12 | -2.07 | -2.42 |
| EW997084.1 | endonuclease g | 403 | XP_001892352 | 2.20E-07 | -1.95 | -2.14 |
|  |  |  |  |  |  |  |
| Chromatin organisation | |  |  |  |  |  |
| CK149194.1 | chromodomain helicase dna binding protein 5 | 704 | XP_002434698 | 7.08E-57 | -1.29 | -1.32 |
| EW997480.1 | histone h2b | 630 | XP_001609700 | 2.84E-06 | -1.46 | -1.76 |
|  |  |  |  |  |  |  |
| Misc |  |  |  |  |  |  |
| CV548308.1 | alkaline phosphatase | 403 | AAV69062 | 2.96E-12 | -1.09 | -1.46 |
| DW474413.1 | angiotensin converting enzyme | 567 | XP_418074 | 9.45E-63 | -1.15 | -1.47 |
| CV548235.1 | MAM domain containing protein | 541 | XP_002215121 | 2.43E-14 | -1.80 | -2.41 |
| CK149521.1 | ChaC, cation transport regulator 2 | 470 | NP_001026203 | 1.16E-32 | -1.36 | -1.43 |
| CK656750.1 | ChaC, cation transport regulator 2 | 653 | NP_001025128 | 1.26E-36 | -1.44 | -1.45 |
| CK386793.1 | wd-repeat protein | 157 | XP_002224322 | 4.31E-11 | -1.15 | -1.54 |
| CN655004.1 | glutamine synthetase | 500 | CAD90162 | 8.74E-47 | 1.13 | -1.52 |
| CK149186.1 | flocculin | 618 | XP_002419998 | 9.60E-04 | -1.16 | -1.46 |
|  |  |  |  |  |  |  |
